# Distinct microbes, metabolites, and ecologies define the microbiome in deficient and proficient mismatch repair colorectal cancers

**DOI:** 10.1186/s13073-018-0586-6

**Published:** 2018-10-31

**Authors:** Vanessa L. Hale, Patricio Jeraldo, Jun Chen, Michael Mundy, Janet Yao, Sambhawa Priya, Gary Keeney, Kelly Lyke, Jason Ridlon, Bryan A. White, Amy J. French, Stephen N. Thibodeau, Christian Diener, Osbaldo Resendis-Antonio, Jaime Gransee, Tumpa Dutta, Xuan-Mai Petterson, Jaeyun Sung, Ran Blekhman, Lisa Boardman, David Larson, Heidi Nelson, Nicholas Chia

**Affiliations:** 1 Department of Veterinary Preventive Medicine, The Ohio State University College of Veterinary Medicine, Columbus, OH USA; 2 Microbiome Program, Center for Individualized Medicine, Mayo Clinic, Rochester, MN USA; 3 Division of Surgical Research, Department of Surgery, Mayo Clinic, Rochester, MN USA; 4 Department of Health Sciences Research, Mayo Clinic, Rochester, MN USA; 5 Center for Individualized Medicine, Mayo Clinic, Rochester, MN USA; 6 Department of Genetics, Cell Biology, and Development, University of Minnesota, Minneapolis, MN USA; 7 Division of Laboratory Medicine and Pathology, Mayo Clinic, Rochester, MN USA; 8 Carl R. Woese Institute for Genomic Biology, Department of Animal Sciences, Division of Nutritional Sciences, University of Illinois, Urbana-Champaign, IL USA; 9 Human Systems Biology Laboratory, National Institute of Genomic Medicine, Mexico City, Mexico; 10 Coordinación de la Investigación Científica, Red de Apoyo a la Investigación, UNAM, Mexico City, Mexico; 11 Mayo Clinic Metabolomics Core Laboratory, Mayo Clinic, Rochester, MN USA; 12 Division of Gastroenterology and Hepatology, Mayo Clinic, Rochester, MN USA; 13 Division of Colon and Rectal Surgery, Department of Surgery, Mayo Clinic, Rochester, MN USA

## Abstract

**Background:**

Links between colorectal cancer (CRC) and the gut microbiome have been established, but the specific microbial species and their role in carcinogenesis remain an active area of inquiry. Our understanding would be enhanced by better accounting for tumor subtype, microbial community interactions, metabolism, and ecology.

**Methods:**

We collected paired colon tumor and normal-adjacent tissue and mucosa samples from 83 individuals who underwent partial or total colectomies for CRC. Mismatch repair (MMR) status was determined in each tumor sample and classified as either deficient MMR (dMMR) or proficient MMR (pMMR) tumor subtypes. Samples underwent 16S rRNA gene sequencing and a subset of samples from 50 individuals were submitted for targeted metabolomic analysis to quantify amino acids and short-chain fatty acids. A PERMANOVA was used to identify the biological variables that explained variance within the microbial communities. dMMR and pMMR microbial communities were then analyzed separately using a generalized linear mixed effects model that accounted for MMR status, sample location, intra-subject variability, and read depth. Genome-scale metabolic models were then used to generate microbial interaction networks for dMMR and pMMR microbial communities. We assessed global network properties as well as the metabolic influence of each microbe within the dMMR and pMMR networks.

**Results:**

We demonstrate distinct roles for microbes in dMMR and pMMR CRC. *Bacteroides fragilis* and sulfidogenic *Fusobacterium nucleatum* were significantly enriched in dMMR CRC, but not pMMR CRC. These findings were further supported by metabolic modeling and metabolomics indicating suppression of *B. fragilis* in pMMR CRC and increased production of amino acid proxies for hydrogen sulfide in dMMR CRC.

**Conclusions:**

Integrating tumor biology and microbial ecology highlighted distinct microbial, metabolic, and ecological properties unique to dMMR and pMMR CRC. This approach could critically improve our ability to define, predict, prevent, and treat colorectal cancers.

**Electronic supplementary material:**

The online version of this article (10.1186/s13073-018-0586-6) contains supplementary material, which is available to authorized users.

## Background

The gut microbiota has been linked to colorectal cancer (CRC) in many studies [1–9] and serves as a very promising target for diagnostic, prophylactic, and therapeutic applications. Yet, despite intense study, only a few microbial species—like *Fusobacterium* species—are consistently observed across studies [10–14], while many microbial associations appear to be cohort-specific. Meta-analyses have attempted to overcome the limited statistical power of smaller studies [15] but are limited by the strong biases introduced through varying collection, sequencing, and data processing methodologies [16]. Mechanistic studies in mouse models have identified strong causative links between specific microbes (e.g., *Fusobacterium nucleatum*, *Bacteroides fragilis*) and CRC development and progression [11, 17–24], but these models have limited applicability in genetically diverse human populations. Capturing some of this genetic diversity, on the other hand, may improve our ability to discriminate tumor and normal microbial communities and more clearly define pathways to CRC.

One genetic subtype of CRC is based on the presence or absence of mutations in the DNA mismatch repair system. This system involves multiple protein complexes that recognize, remove, and correct mismatched DNA base pairs. Mutations in these protein complexes can render the mismatch repair system defunct—allowing mutations to accumulate. This hypermutable subtype is known as deficient mismatch repair (dMMR) and occurs in approximately 15% of sporadic CRCs [25]. CRCs that do not exhibit mutations in the mismatch repair system are known as proficient mismatch repair (pMMR) CRCs [26]. In general, dMMR CRCs are microsatellite instable (MSI-H), hypermethylated, and associated with BRAF V600E mutations and low nuclear beta-catenin expression, whereas pMMR CRCs are more commonly microsatellite stable (MSS) and associated with KRAS mutations [27, 28]. Clinically, MMR status is associated with patient prognosis and age, as well as tumor location and stage: Specifically, dMMR CRCs have a better prognosis and occur more often on the right side of the colon in older patients with early-stage CRC [26]. Finally, dMMR and pMMR CRC not only have different endpoints, but may also have different paths to tumorigenesis [29] as supported by emerging evidence that dMMR CRC arises from sessile serrated adenomas [30] as opposed to the more classic tubular adenoma associated with pMMR CRC [30].

The distinct phenotype of dMMR CRC suggests that host—and possibly also microbial—dynamics are greatly altered in association with deficient mismatch repair. Previous work has examined the role of other differentiating factors in the CRC microbiome including location [31], MSS/MSI status [12], and consensus molecular subtypes [32]. However few CRC microbiome studies account for MMR status [32–34] or microbial dynamics [35], and no studies, to our knowledge, have assessed both MMR status and microbial community dynamics. In addition, our study examines demographic, genetic, and tumor features together in a relatively large prospectively collected cohort.

Here, we undertook a new approach in a study involving 83 patients who underwent partial or total colectomy for CRC. From each patient, we collected colon tissue and mucosal samples at tumor and normal-adjacent sites. MMR status was extracted from patient records or determined by testing formalin-fixed paraffin-embedded tumor tissue for the expression of four MMR proteins (MLH1, MSH2, MSH6, PMS2). From this, patient tumors were characterized as either deficient (dMMR) or proficient (pMMR) mismatch repair. Microbial composition was assessed via 16S rRNA gene sequencing. A subset of colon tissue samples additionally underwent targeted metabolomic analysis to quantify amino acids. A portion of these data was published previously [35] in a study that highlighted the value of integrating in silico genome-scale metabolic model predictions and in vivo experimental metabolomic data.

From these data, we assessed the relative importance of MMR status compared to other biological factors reported to alter the microbiome [36]. MMR status was the strongest predictor of microbial community variance in comparison to sample location (proximal/distal and on/off tumor), body mass index (BMI), age, and sex. Separate analyses of the dMMR and pMMR microbial communities revealed that many common CRC-associated microbial signatures [15]—including *Fusobacterium nucleatum*, *Fusobacterium periodonticum*, and *Bacteroides fragilis—*were all enriched in dMMR but not pMMR tumors. Functional differences were examined using a combination of metabolomics and community metabolic modeling. Our results indicate greater hydrogen sulfide production (inferred through amino acid proxies) in dMMR CRC and greater metabolic suppression of *B. fragilis* in pMMR CRC. Our work demonstrates distinct microbial, metabolic, and ecological attributes of dMMR and pMMR microbial communities, serving to further emphasize the importance of considering tumor biology and microbial interactions in studies of the CRC microbiome.

## Methods

### Human subject enrollment

Adults (older than 18 years old) who were determined to be candidates for colorectal cancer surgery were voluntarily enrolled at Mayo Clinic in Rochester, Minnesota. Exclusion criteria included chemotherapy or radiation in the 2 weeks leading up to enrollment. Total or partial colectomies were performed on every patient, and colon tissue and mucosal samples were collected from tumor and normal-adjacent sites. Sample location was defined as follows: “proximal” samples were derived from the cecum and ascending colon. “Distal” samples were derived from the transverse, descending, or sigmoid colon, or rectum. MMR status was determined in 83 patients: 25 had dMMR CRC and 58 had pMMR CRC (Table 1). We used univariable logistic regression (R v3.1.2) to compare demographic (age, sex, BMI, smoking history) and tumor features (location and stage) between dMMR and pMMR groups.

**Table 1:** Demographic and tumor features of individuals identified as having dMMR or pMMR CRC

	dMMR	pMMR	*p* value
Sex, *n* (%)			
Male	10 (40)	34 (59)	0.122
Female	15 (60)	24 (41)	
Age, years			
Mean (SD)	74 (18)	63 (13)	0.002
Range	23–95	33–90	
BMI (SD)	27 (5)	29 (8)	0.273
Smoke ever? *n* (%)			
Yes	13 (52)	28 (48)	0.982
No	12 (48)	30 (52)	
Tumor location, *n* (%)			
Proximal colon	18 (72)	14 (24)	*p* < 0.0001 between proximal and distal
Distal colon	7 (28)	43 (74)	
Both	0	1 (3)	
Stage, *n* (%)			
Early (1–2)	18 (72)	22 (38)	0.0007 between early and late
Late (3–4)	4 (16)	33 (57)	
Stage unknown	3 (12)	3 (5)	

### MMR status determination

Mismatch repair (MMR) pathway and microsatellite instability (MSI) test results were extracted from patient records if available. For patients without MMR test results, banked formalin-fixed paraffin-embedded colon tumor tissue blocks were submitted to the Mayo Clinic Pathology Resource Core for sectioning into 10-μm-thick slices. Slices were then submitted to the Mayo Clinic Molecular Genetics Laboratory for immunohistochemistry staining of MMR proteins (MLH1, PMS2, MSH2, MSH6).

### 16S DNA extraction, sequencing, and sequence processing

DNA extraction [37] and library preparation on colon tissue (tumor and normal-adjacent) and mucosa were performed as described previously in the Mayo Clinic Microbiome Laboratory [35]. Samples were submitted for 16S rRNA gene sequencing (V3–V5 region) at the Mayo Clinic Medical Genomics Facility (Illumina MiSeq, 2 × 300, 600 cycles, Illumina Inc.). Sequencing yielded a total of 41,400,384 reads with a median of 70,208 reads per sample. Reads were processed using DADA2 v1.6 to obtain error-corrected amplicon sequence variant representatives—analogous to operational taxonomic units with single-nucleotide resolution (sOTUs) [38]. sOTUs were annotated with genus-level taxonomy using the RDP Naïve Bayesian Classifier [39] as implemented in DADA2 and, if possible, to species level using DADA2, both against the SILVA 16S database, v132 [40]. sOTUs annotated as chloroplast and mitochondria were removed. Resulting sOTUs were filtered for possible non-specific amplification using SortMeRNA v2.0 [41] and Infernal v1.1.2 [42]. sOTUs with fewer than 10 reads across all samples were excluded. Multiple sequence alignment of the sOTUs was performed using Infernal v1.1.2 [42], and an approximate Maximum Likelihood phylogeny was calculated using FastTree v2.1.9 [43].

### Statistical analyses of 16S rRNA microbial community data

UniFrac distance matrices [44] based on the microbial communities in all samples were generated using the phyloseq [45] package v1.22.3. A permutational multivariate analysis of variance (PERMANOVA) was then performed on the distance matrix to assess the effects of MMR status and sample location (proximal/distal and on/off tumor) on variance between microbial communities. The PERMANOVA additionally accounted for subject age, sex, BMI, and sample type (mucosa versus colon tissue) and was performed based on the adonis function in the vegan [46] package v2.5-1, with 999 permutations. Different permutation schemes were used to maintain the original correlation structure when testing the significance of relevant variables.

A generalized linear mixed model (GLMM) [47] was calculated for each sOTU to estimate its abundance (read counts) in relation to predictors that included MMR status and sample location (proximal/distal and on/off tumor). Models were corrected for subject intervariability, specimen type (mucosal vs tissue biopsy), and sequencing read depth, allowing for interactions. We used the package glmmTMB [48] v0.1.4 to estimate the abundance of each microbe under a zero-inflated Poisson distribution. For each predictor, sOTUs were excluded where the method did not converge or the Akaike Information Criterion (AIC) for model quality was not defined. Multiple hypothesis correction was calculated using the Benjamini–Hochberg procedure.

### Validation of differentially abundant microbes using an independent cohort

To validate the differentially abundant microbes associated with dMMR status, we investigated data from a recent study that included microbiome profiling in tumor and matched normal tissue samples in 44 CRC patients [49]. Individuals with microsatellite instable (MSI-H) tumors or downregulation of any of the 4 MMR genes (MLH1, MSH2, MSH6 and PMS2)—as assessed using RNA-Seq—were categorized as dMMR. A cutoff of log2(normal/tumor) ≥ 1 was used to call a gene as downregulated in tumor. Individuals with microsatellite stable (MSS) tumors were categorized as pMMR. Altogether, we identified 9/44 patients as dMMR and the remaining 35/44 as pMMR. Using the 16S rRNA gene to characterize these samples (as described in detail in [49]), we identified sOTUs associated with dMMR tumor/normal and pMMR tumor/normal conditions. We first filtered rare sOTUs, only preserving sOTUs found in at least 50% of our samples, and then performed differential abundance analysis using phyloseq [45] (which uses DESeq2 to build negative binomial generalized linear models). We used the Benjamini–Hochberg method to control for the false discovery rate (FDR).

### Real-time PCR for the *Bacteroides fragilis* toxin gene

Real-time PCR was performed as described previously [35] to test colon tissue and mucosal samples for the presence of the *Bacteroides fragilis* toxin (BFT) genes in the 22 dMMR individuals and 53 pMMR individuals. Primers included: BFT-F (5′-GGATAAGCGTACTAAAATACAGCTGGAT-3′), BFT-R (5′-CTGCGAACTCATCTCCCAGTATAAA-3′), and the probe (5′-FAM-CAGACGGACATTCTC-NFQ-MGB-3′) [19].

### Modeling microbial hydrogen sulfide production

We predicted hydrogen sulfide production within dMMR and pMMR tumor and normal-associated microbial communities as described previously [35]. Briefly, we aligned 16S rRNA gene sequences for dMMR tumor and normal samples (colon tissue and mucosa) and pMMR tumor and normal samples against complete genomes in PATRIC and then generated genome-scale metabolic models of each microbe (Additional file 1: Table S1). Genome-scale metabolic models use gene annotations from a microbial genome to predict the metabolic inputs and outputs of that microbe. To predict how a microbe might interact within a community, we used MICOM, an open-source platform to assess microbial community metabolism (https://github.com/resendislab/micom). Specifically, we used flux balance analysis with MICOM’s community growth objective and constraint formulation in order to evaluate hydrogen sulfide flux as a measure of hydrogen sulfide production within each microbial community.

### Microbial influence network

To select sOTUs for the Microbial Influence Networks (MINs), we used GLMM results to choose tumor and normal-associated microbes in dMMR and pMMR samples with a linear effect size greater than 0.25, regardless of statistical significance. Effect size captures biological impact potential while significance measures certainty. In this case, we wanted to assess the metabolic influence (i.e., biological impact) of microbes in relation to their respective microbial communities; as such, it was more appropriate to filter by effect size. For each sOTU, the 16S rRNA gene consensus sequence was aligned against complete genome in the PATRIC system using VSEARCH v2.7.1, with a minimum nucleotide identity of 90%. When this procedure generated multiple top hits, we selected a genome, in order, to the most complete genome (fewer contigs), a type strain, a strain with a binomial name, and the closest match to the 16S taxonomy (when possible). For each genome, we then reconstructed and downloaded its corresponding genome-scale metabolic model using the PATRIC service. When sOTUs mapped to the same model, we used that model only once, effectively merging those sOTUs in further analysis, with an exception for when two sOTUs were associated with opposite conditions (i.e., tumor and normal-adjacent samples), in which case, we discarded that model from further consideration. The decision to discard was also based on the observation that low identity hits or sOTUs with taxonomy not sufficiently resolved were typically involved in these few cases.

After obtaining the genome-scale metabolic models (GEMs), we calculated “growth” on complete media with no oxygen. This was done by calculating optimal metabolic reaction fluxes using a Flux Balance Analysis [50], in which “growth” is the calculated flux of the reaction defining biomass for a microbe. We did this using a tool for assessing microbial metabolic interactions (MMinte) which evaluates the growth of microbes alone and when paired with another microbe [51]. Once single and paired growth values were calculated using the objective function given by MMinte [51], these values were then used to calculate the influence score. The influence score, *α*_xm_, for a species, m, with a different species x was calculated as

1 $$ {\alpha}_{xm}=g\left(x|m\right)-g(x) $$

where *g*(x) was the growth rate of *x* alone and *g*(x| *m*) was the growth rate of *x* in a community composed of both x and m. Based on these scores, we then calculated the influence of each individual microbial model on the other microbes in the community as the sum of the absolute values of the differences in growth rates when paired with species m,

2 $$ {G}_m=\sum \limits_j\left|{\alpha}_{jm}\right|=\sum \limits_j\left|g\left(j|m\right)-g(j)\right| $$

This scoring closely follows the spirit of the scoring from the global metabolic interaction modeling in Sung et al. [52]; to derive interactions, we used growth rates that were computationally inferred from comprehensive metabolic models in contrast to using experimentally verified transport reactions from a limited number of microbes and metabolites. Metabolic modeling based on flux balance analysis, as described here, provides a means to calculate a rate of steady-state growth, as normalized per unit mass, allowing us to take a simple sum in order to calculate influence under anaerobic conditions.

The percentage of negative interactions was calculated by counting the number of negative interactions over the number of total interactions in each microbial influence network (MIN). Statistical significance was based on the probability of getting equivalent results in dMMR and pMMR networks using the measured distributions of negative and positive interactions in each network and a scheme of random selection with replacement.

Finally, the resulting MIN [52] was visualized using Cytoscape v3.6.1 [53] with node size and edge weights set according to influence score and influence, respectively. The entire list of microbial influences in dMMR and pMMR subjects (Additional file 1: Tables S8 and S9) are too dense for direct visualization, and therefore, only a part of them are presented. More specifically, interactions below an influence of 10 in the case of both dMMR and pMMR were excluded. Unconnected nodes that had no influence were not included in the visualization.

### Estimating the effect of whole-community metabolic interactions on growth suppression

In order to assess the degree to which a microbial species is suppressed by other members of the microbial community, we evaluate the interactions of the rest of the microbial community on a target member. It is worth emphasizing that this infers the effect of all members of a microbial community, in contrast to the MIN, which focuses on the tumor and normal-adjacent enriched microbes in dMMR and pMMR CRC. Briefly, the target organism’s net interactions *S*_*m*_ with each microbe in a given community will be calculated according to:

3 $$ {S}_m=\sum \limits_j{A}_j{\alpha}_{mj}=\sum \limits_j{A}_j\left(g\left(m|j\right)-g(m)\right) $$

i.e., the abundance-weighted sum of the metabolic influences on microbe *m*. When this sum is negative (as would be generally true in eubiosis), this yields a suppression score that reflects the magnitude of the negative interactions affecting microbe m. For the purpose of this calculation, we calculate this in every sample, we use anaerobic conditions and only consider microbial species that make up greater than 5% of the relative abundance of the community in at least one sample, ensuring we do not miss any microbes that may have a significant effect on the suppression score.

## Results

### dMMR tumors associated with older-age and early-stage, proximal tumors

A total of 25 individuals with dMMR CRC and 58 individuals with pMMR CRC were involved in this study. Individuals with dMMR CRC were significantly older than individuals with pMMR CRC and significantly more likely to have an early-stage, proximal tumor (Table 1)—in alignment with other studies on dMMR CRC [25]. Thus, to address potential confounding effects due to age and sample location (proximal/distal), we adjusted these variables in subsequent analyses.

### Tumor MMR status explains the largest variance between microbial communities

To assess factors that contributed to variance in the microbial community data, we performed a PERMANOVA analysis on unweighted UniFrac distances between microbial communities in each sample. We included MMR status, sample location (proximal/distal, on/off tumor), age, sex, BMI, and sample type (colon tissue vs. mucosa) as potential predictors of the variance. We used both marginal and adjusted analyses where we included only a single factor in our assessment of percent variance explained or after correcting for all other factors, respectively. The adjusted analysis controlled the effects of other variables, and the resulting percent variance explained was independent of other variables and thus not subject to the confounding by the correlated variables. Remarkably, we found that MMR status explained more of the variance than any of the other 6 variables in both cases (Table 2), and it remained significant after adjusting for other variables (*p* = 0.004), indicating MMR status was independently associated with the microbiome composition. The difference between tumor and normal-adjacent samples was also highly significant (*p* < 0.001 from adjusted analysis; Table 2), indicating that the tumor samples harbor a unique microbiome. Moreover, when comparing the tumor-to-normal UniFrac distance between MMR subtypes (Additional file 1: Figure S1), the distance in the dMMR subtype was significantly larger than that in the pMMR subtype (*p* = 0.004), which suggests a potential stronger perturbation of the normal microbiome in the dMMR subtype.

**Table 2:** Factors contributing to variance between microbial communities

	Marginal		Adjusted	
Factors	% Variation	*p* value	% Variation	*p* value
MMR status	2.58	0.001	1.85	0.004
Sample location—proximal/distal	1.87	0.011	1.44	0.019
Sample type	1.70	0.001	1.36	0.001
Sample location—on/off tumor	1.50	0.001	1.01	0.001
Sex	1.48	0.051	1.14	0.184
BMI	1.34	0.108	1.60	0.025
Age	0.96	0.542	1.02	0.319

Percent variation and *p* values in the first two columns were from marginal analyses (i.e., not adjusted for other factors). Percent variation and *p* values in the last two columns were from analyses adjusting for all factors. Permutation tests (999 permutations) were used to calculate the *p* values. For “Sample type” and “on/off tumor” factors, the permutation was confined within the subject. For the rest of the factors, the subjects were the permutation units (i.e., randomly assign a value to each subject) to account for the within-subject correlations

### Distinct microbial communities associated with pMMR and dMMR tumors

**Fig. 1:**
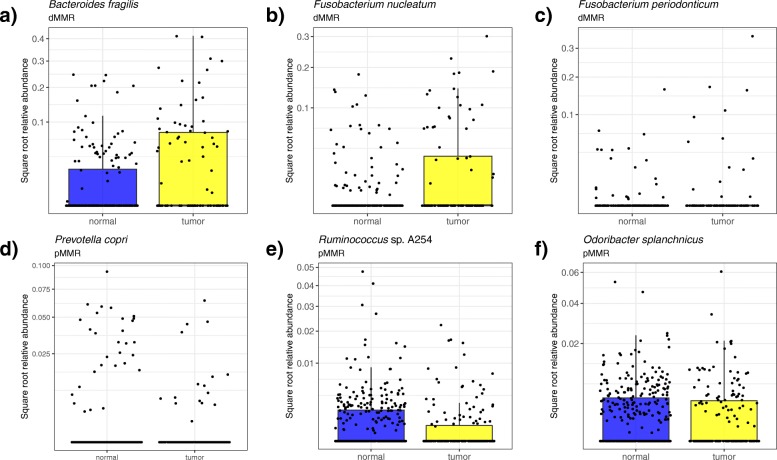
Top 3 microbes significantly enriched in tumor as compared to normal samples (colon tissue and mucosa) in individuals with (**a**, **b**, **c**) dMMR or (**d**, **e**, **f**) pMMR CRC. For full results, please see Additional file 1: Tables S2-S4. Notably, the top 3 microbes enriched in dMMR CRC tumor samples were not enriched at all in pMMR CRC and vice versa. *Y*-axis is square root transformed. See Additional file 1: Figure S3 for stratification of these results by tumor location

Given both the importance of MMR status to microbial community variance (Table 2) and the difference in tumor to normal UniFrac distances by MMR subtype (Additional file 1: Figure S1), we opted to assess microbial abundances in tumor and normal samples for each MMR subtype independently. We identified multiple differentially abundant sOTUs in dMMR and pMMR tumor samples as compared to normal-adjacent samples using a generalized linear mixed model (GLMM) that accounted for sample location (proximal/distal), sample type, and intrasubject sample correlation (Fig. 1; Additional file 1: Figure S2 (Venn diagram showing counts of microbes in each group); Additional file 1: Table S2 (list of microbes enriched in dMMR and pMMR tumor and normal-adjacent samples); Additional file 1: Table S3 (microbes enriched in dMMR tumors); Additional file 1: Table S4 (microbes enriched in pMMR tumors); Additional file 1: Table S5 (microbes enriched in the proximal or distal colon of individuals with dMMR CRC); Additional file 1: Table S6 (microbes enriched in the proximal or distal colon of individuals with pMMR CRC)). Only one microbe—*Dorea longicatena*—was significantly enriched in both dMMR and pMMR tumor samples. Four microbes had opposite associations with tumor or normal samples depending on MMR status: *Faecalibacterium prausnitzii* A2-165 and *Blautia* sp. Marseille-P2398 were significantly enriched in pMMR tumor and dMMR normal samples; *Coprococcus comes* ATCC 27758 and *Bacteroides massiliensis* B84634 were significantly enriched in dMMR tumor and pMMR normal samples. Notably, *Fusobacterium* and *Bacteroides fragilis*—microbes commonly associated with CRC [11, 17–24]—were among the top most differentially abundant microbes in dMMR tumor samples but were not found to be differentially abundant in pMMR tumor samples. As the GLMM had adjusted sample type, sample location (distal/proximal), and age in the model, these significant associations were less likely to be driven by these potential confounders. Indeed, we observed an enrichment of the dMMR-associated microbes regardless of sample locations (Additional file 1: Figure S3).

To validate these results, we used publicly available data from tumor and matched normal samples from 44 CRC patients [49]. Our validation analysis showed several overlapping associations of microbial genomes with respect to dMMR and pMMR in tumor and matched normal samples (Additional file 1: Tables S7, S8). dMMR tumors were found enriched for *B. fragilis* (*p* = 0.02, FDR *p* = 0.37) and *Fusobacterium* (*p* = 0.03, FDR *p* = 0.37) while dMMR normal samples were enriched for *Dorea* (*p* = 0.03, FDR *p* = 0.37) and an Erysipelotrichaceae bacterium (*p* = 0.007, FDR *p* = 0.31) (Additional file 1: Figure S4). Even though these associations were not statistically significant after correcting for FDR, their trend of association overlaps with the results from the present study. Differentially abundant sOTUs between pMMR tumors versus normal included Ruminococcaceae, *Faecalibacterium prausnitzii*, and *Bacteroides caccae*, which were also differentially abundant in the present study.

### Proxies for hydrogen sulfide production enriched in the dMMR CRC tumors

**Fig. 2:**
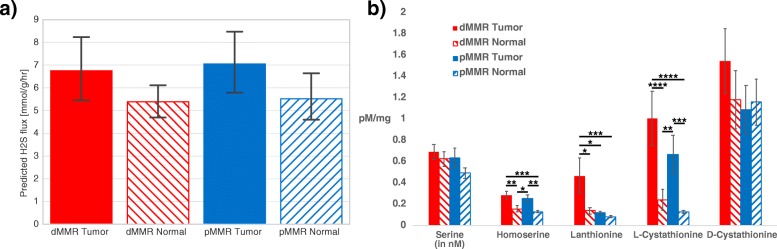
**a** Hydrogen sulfide flux predicted based on community metabolic modeling. Flux was predicted in millimol per gram dry weight of bacteria per hour. **b** Amino acid proxies for hydrogen sulfide were quantified using UPLC-MS on dMMR and pMMR tumor and normal-adjacent colon tissue samples (Kruskal-Wallis followed by Dunn’s Test for posthoc comparisons: *p < 0.05; **p < 0.0005, ***p < 0.0005, ****p < 0.00005)

As sulfidogenic *F. nucleatum* and *F. periodonticum* were also significantly enriched in dMMR tumor samples, we decided to assess potential hydrogen sulfide production across groups (dMMR/pMMR, tumor/normal) by modeling hydrogen sulfide flux. We used microbial community metabolic models to predict hydrogen sulfide flux within each microbial community using MICOM. We then took the values for the hydrogen sulfide flux and calculated the average value within each group (dMMR tumor and normal, pMMR tumor and normal). The models produced a non-significant trend towards increased hydrogen sulfide flux in tumor samples (Fig. 2a). To get a more concrete measure of hydrogen sulfide production, we ran targeted metabolomics to quantify amino acid proxies (serine, homoserine, lanthionine, l-cystathionine, d-cystathionine) for hydrogen sulfide in dMMR and pMMR tumor and normal tissue samples (Fig. 2b). We observed a significant increase in lanthionine in dMMR tumor tissue over dMMR or pMMR normal tissue and pMMR tumor tissue. Homoserine and l-cystathionine were also significantly increased in both dMMR and pMMR tumor tissue as compared to normal-adjacent tissue. The metabolomics results suggest increased hydrogen sulfide production in tumor tissue—particularly in dMMR tumor tissue.

### dMMR and pMMR tumor and normal-adjacent microbial community predicted to be highly influenced by differing Bacteroides species

**Fig. 3:**
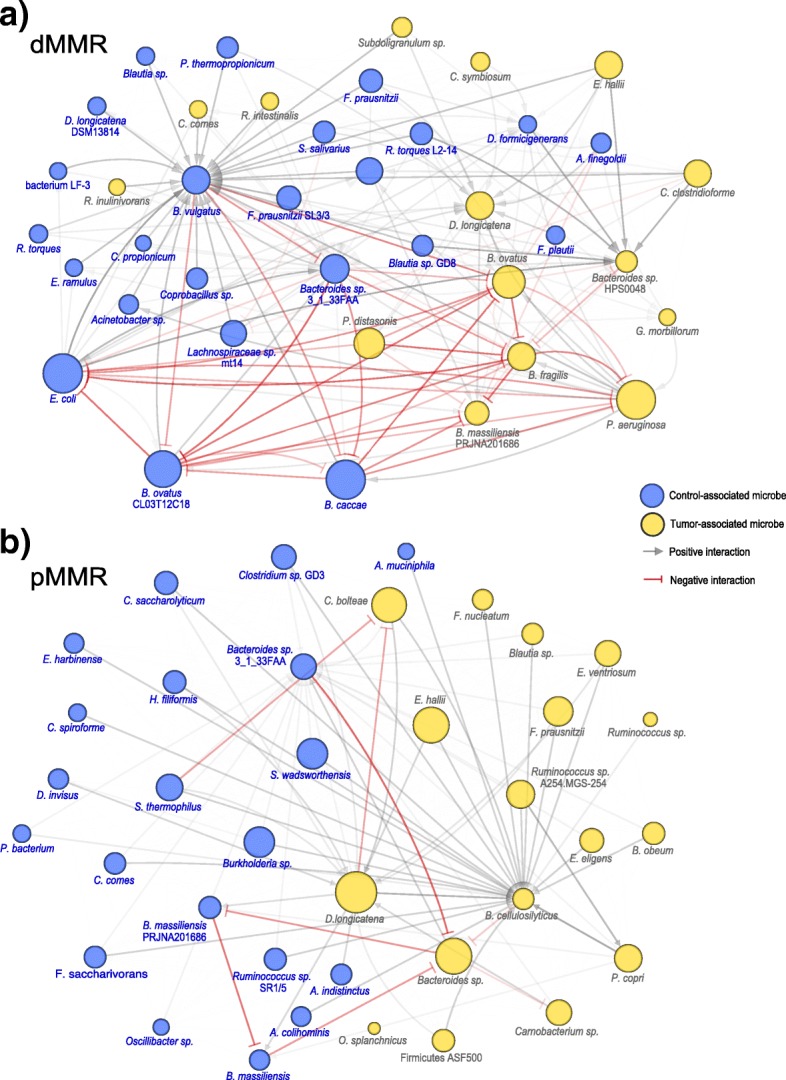
Microbial influence networks for **a** dMMR and **b** pMMR microbial communities. Node size indicates a microbe’s metabolic influence over other microbes. Edges, which are directional and weighted according to the magnitude of their influence, indicate how one microbe affects the growth rate of another. Grey edges indicate a positive interaction, i.e., predicted increase in growth when paired, while red edges indicate a negative interaction, i.e., predicted suppression in growth when paired

To further assess the potential metabolic interactions between tumor and normal-adjacent microbes in relation to MMR status, we constructed two metabolic influence networks (MIN; Fig. 3) [52]. The MIN highlights each microbe’s predicted influence and interactions (growth enhancing or suppressing) in relation to other microbes in the community. The major influencers (largest nodes) are generally composed of primary fermenters, such as *Bacteroides* or *Prevotella*. However, in dMMR, the normal-adjacent community is anchored by the highly influential *B. caccae* and *B. ovatus* while in the pMMR MIN, the tumor community is anchored by *D. longicatena* and a *Bacteroides* sp. These different dMMR and pMMR communities appear to have different key species that influence the rest of the microbial community. Also of note in relation to the dMMR MIN, *F. nucleatum* and *F. periodonticum* exhibit no metabolic interactions with the other microbes in the network and therefore were not included in the network visualization.

### pMMR microbial community predicted to enhance suppression of *Bacteroides fragilis*

**Fig. 4:**
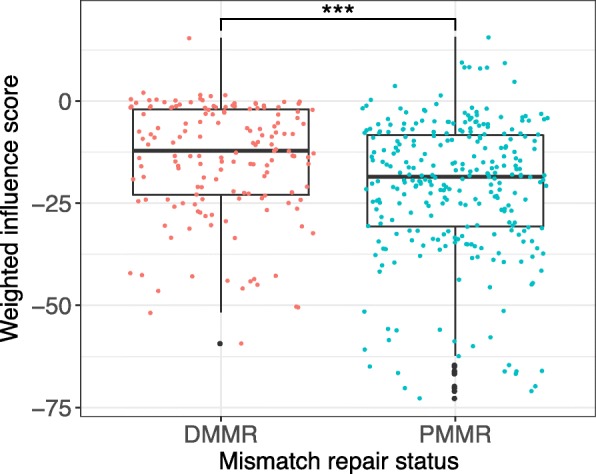
Predicted influence of other microbes (weighted influence score) on *B. fragilis* growth, stratified by MMR status. A negative influence score indicates microbial community suppression of *B. fragilis* growth. *B. fragilis* is significantly more suppressed in pMMR microbial communities (tumor and normal) as compared to dMMR microbial communities (Wilcoxon rank sum test *p* < 0.001)

The differences between the MINs for dMMR and pMMR microbial communities only relate to the tumor or normal-adjacent associated microbiota. While remarkable and noteworthy for understanding how the key species metabolically suppress or promote one another, it is nonetheless an incomplete picture of the effect of the microbial community on bacterial species growth. In order to better assess the impact of dMMR and pMMR communities as a whole on *B. fragilis*, we computed an interaction score and took an abundance-weighted sum of the effect of those interactions of *B. fragilis* for each microbial community. When comparing dMMR and pMMR communities, we see a statistically significant difference (Fig. 4; Wilcoxon rank sum *p* < 0.001) in the growth suppression of *B. fragilis*, with markedly more suppression in pMMR communities where *B. fragilis* is not associated with CRC. This is consistent with the idea that *B. fragilis* may play a central role in dMMR but not pMMR CRC as suggested by our GLMM.

Given this finding and the well-established links between toxigenic *B. fragilis* and colorectal cancer [19, 21, 24], we next looked for the presence of the *B. fragilis* toxin (BFT) gene in dMMR and pMMR tissue and mucosa samples. Of the 22 individuals with dMMR CRC, only one was BFT positive (5%); of 53 individuals with pMMR CRC, only five were BFT positive (9.4%). There was no significant difference in BFT presence between individuals with dMMR or pMMR CRC (Chi-squared, *p* = 0.477).

## Discussion

This study integrates tumor biology and microbial ecology in a novel and powerful approach to understanding colorectal cancer. Our results indicate that MMR status is one of the strongest predictors of microbial community variance; however, few studies [32–34], to date, include MMR status in microbial community analysis of colorectal cancer. Interestingly, we also identified several differentially abundant microbes associated with dMMR but not pMMR tumor samples including *F. nucleatum*, *F. periodonticum*, and *B. fragilis.* We further validated these findings in an independent cohort [49], which underscores the importance of including MMR status in future CRC microbiome studies. We additionally characterized the predicted and actual metabolic profiles of dMMR and pMMR individuals in relation to hydrogen sulfide production, and we generated a network of predicted interactions within the dMMR and pMMR microbial communities.

Hydrogen sulfide has been reported to both promote and inhibit colorectal cancer [54–57]. To assess the role of hydrogen sulfide within our study, we looked for sulfidogenic bacteria, predicted hydrogen sulfide production using community metabolic models, and indirectly measured hydrogen sulfide concentrations through targeted metabolomics for amino acid proxies. We found two significantly enriched hydrogen sulfide-producing *Fusobacterium* species and significantly increased proxies for hydrogen sulfide in dMMR tumor samples. In the microbial influence network, both *Fusobacterium* species exhibited zero predicted interactions—positive or negative—with other microbes in the network. Together, this suggests that these *Fusobacterium* species may grow unchecked by other microbes and have the potential to produce large quantities of hydrogen sulfide.

These intriguing results lead us to speculate on the relationship between *Fusobacterium* species, hydrogen sulfide production, and dMMR CRC. Notably, *Fusobacterium* species have previously been associated with hypermethylation of MLH1, MSI, BRAF mutations, and poorly differentiated tumors [12, 22]—all of which are characteristics of dMMR CRC [25]. Hydrogen sulfide—a cytotoxic, genotoxic gas—has also been associated with CRC [54, 55], although there have been conflicting reports on its role [56, 57]. A recent report indicates that colon cancer cells may respond to hydrogen sulfide in a bell-shaped dose-dependent manner: at high concentrations, hydrogen sulfide inhibits the proliferation of cancer cells, while at lower concentrations, hydrogen sulfide can stimulate the proliferation of cancer cells [57, 58]. In dMMR, if high levels of hydrogen sulfide (and hydrogen sulfide producers) inhibit cancer proliferation, then we would expect individuals with dMMR to present with earlier-stage cancer—which is indeed the case in our cohort and other reported cohorts [25].

Epidemiologically, it is worth noting that dMMR CRC has also been associated with lower recurrence rates and a better prognosis [25]. In seeming opposition to these findings are studies showing that *F. nucleatum* can potentiate tumorigenesis and that *F. nucleatum*-associated CRCs have a worse prognosis [11, 12]. However, these findings are not contradictory with our data. A more detailed examination of the effects of location (Additional file 1, Tables S5 and S6) shows that *Fusobacterium* is associated with the proximal colon in both dMMR and pMMR patients. This raises a subtle, but important, point. *Fusobacterium*-associated pMMR tumors are very likely to be found in the proximal colon alongside normal-adjacent tissue that is also enriched for *Fusobacterium*. Stated another way, while pMMR tumors are not especially associated with *Fusobacterium*, the proximal colon is. (In contrast, dMMR tumors show enrichment for *Fusobacterium* that goes beyond the effect of location in the colon.) When put into context with other epidemiological findings that identify right-sided (proximal) colon cancer to have lower overall survival [59], certain inferences come to light. Where right-sided dMMR CRCs have a relatively better prognosis, right-sided pMMR CRCs have a worse one. This would then allow us to make sense of both the overall lower survival in right-sided CRC [59] and the results indicating *F. nucleatum*-associated CRCs have a worse prognosis [11, 12]. In sum, the prognosis of *F. nucleatum*-associated CRCs is likely be dependent upon both location and tumor MMR status, and our study highlights the importance of evaluating these covariates simultaneously when determining tumor prognosis.

Besides *Fusobacterium*, *B. fragilis* was also found to be significantly enriched in dMMR tumor samples. Toxigenic *B. fragilis* has well-established and causative links to inflammation and CRC [19, 21, 24], and inflammation has been linked to hypermethylation [60]. Our own metabolic modeling reflects a metabolic basis for higher ratios of *B. fragilis* in dMMR communities, and greater metabolic suppression in pMMR. We tested dMMR and pMMR tissue and mucosa samples for the presence of the *B. fragilis* toxin (BFT) gene but did not find a significant difference in the presence of the BFT gene between dMMR and pMMR individuals. Given these results, it is unclear what the significance of toxigenic *B. fragilis* is in the dMMR tumor samples.

Overall, our study demonstrates the importance and value in considering tumor biology (MMR status) and ecological interactions when evaluating microbial community data. Our work is primarily descriptive and incorporates host clinical features, microbiome, metabolome, and modeling data. While we make speculations based on these data, future prospective and mechanistic studies are needed to test these ideas. We also recognize that selecting sequenced genomes available in the database to represent 16S rRNA sOTUs cannot fully replace metagenomic sequencing given well-known strain-to-strain variation in gene content. However, these variations between strains are often largely in secondary metabolite pathways, rather than core metabolic function, which is the main target of our modeling analysis. Differences in secondary metabolite pathways (i.e., non-core genome within a species) are commonly associated with functional adaptations to various environmental niches [61].

Another limitation of this study is our inability to attribute a source to metabolomic data. Hydrogen sulfide and its amino acid proxies can be produced by both humans and bacteria. Thus, the enriched hydrogen sulfide we detect in dMMR tumor samples could potentially be attributed to increased hydrogen sulfide production within tumor tissue, and indeed, this has been reported [57]. If this was the solely case here however, we might expect to see similar increases in hydrogen sulfide in pMMR tumors—most of which are later in stage than dMMR tumors. We did not see this, suggesting that it is feasible that the increased hydrogen sulfide production in dMMR tumors is coming from an exogenous (microbial) source. Notably, microbially produced hydrogen sulfide can be generated from multiple pathways including the respiration of dietary taurine and sulfate as well as the degradation of sulfomucins. The amino acid proxies we use to assess hydrogen sulfide production only capture some, but not all of these potential pathways, so we may have underestimated hydrogen sulfide production.

Finally, the field of genome-scale metabolic modeling has only recently encompassed tools for community metabolic analyses [62], and many of the tools [51, 52, 63] are sensitive to the underlying quality of the metabolic models [64, 65]. Models vary greatly depending on the presence and accuracy of genome annotations which will generally improve over time. Future work aimed at understanding and verifying microbial dynamics in relation to MMR status or other CRC subtypes could dramatically improve our ability to define, predict, prevent, and treat colorectal cancers.

## Conclusions

This study provides a novel framework in which to examine colorectal cancer:

1. Host–microbe interactions: Tumor MMR status strongly predicted microbial community variance and was associated with distinct microbial, metabolic, and interaction profiles. Our approach incorporating tumor MMR status, microbiome, metabolome, and modeling data allowed us unique insights into the role of hydrogen sulfide and hydrogen sulfide producers within the dMMR microbial community. Tumor biology (e.g., MMR status) and microbial ecology are inextricably linked, and it is critical that future studies account for both in order to understand and more precisely classify the many pathways to CRC.
2. Microbe–microbe interactions: Microbial influence networks provided in silico predictions of microbial interactions that aligned with in vivo metabolomics data: Enrichment of sulfidogenic *F. nucleatum* and significantly higher hydrogen sulfide production in dMMR CRC, and depletion of *B. fragilis* and significantly higher suppression in pMMR CRC. The validation of in vivo findings and in silico modeling provides support for a future of precision medicine tools that can accurately predict disease and the potential effects of prophylactic or therapeutic interventions on the microbiome. Microbes act within communities, and understanding and predicting these interactions will be key to developing targeted mechanisms to help prevent or treat colorectal cancer.

## Additional files

Additional file 1: **Figure S1.** Unweighted Unifrac distances between tumor and normal-associated microbiota in dMMR and pMMR. **Figure S2.** Venn diagram highlighting number of microbes that overlap between tumor and normal samples in relation to MMR status (dMMR = red font, pMMR = green font, red circles = tumor samples, blue circles =normal samples). **Figure S3.** Microbes significantly enriched in tumor as compared to normal samples (colon tissue and mucosa) in individuals with dMMR CRC: a) *Bacteroides fragilis* and b) *Fusobacterium nucleatum*. **Figure S4.** Differentially abundant OTUs between patient-matched tumor and normal samples in individuals with dMMR CRC from an independent cohort.** Table S1.** **Table S1:** List of PATRIC model IDs and associated sOTUs. **Table S2.** Microbes identified as differentially abundant in tumor as compared to normal samples (tissue and mucosa) from individuals with dMMR or pMMR CRC. **Table S3.** sOTUs enriched in tumor samples (colon tissue and mucosa) as compared to normal adjacent samples in individuals with dMMR CRC. **Table S4.** sOTUs enriched in tumor samples (colon tissue and mucosa) as compared to normal adjacent samples in individuals with pMMR CRC. **Table S5.** sOTUs enriched in the proximal or distal colon (colon tissue and mucosa) of individuals with dMMR CRC. **Table S6.** sOTUs enriched in the proximal or distal colon (colon tissue and mucosa) of individuals with pMMR CRC. **Table S7.** Differentially abundant microbes in individuals with dMMR CRC from an independent cohort. **Table S8.** Differentially abundant microbes in individuals with pMMR CRC from an independent cohort. (PDF 2790 kb)

Additional file 2: sOTU read counts by sample. (TSV 5546 kb)

Additional file 3: sOTU ID and taxonomy. (TXT 654 kb)

Additional file 4: sOTU ID and sequences. (FASTA 2042 kb)
