# Supplementary material for: Distinct microbes, metabolites, and ecologies define the microbiome in deficient and proficient mismatch repair colorectal cancers
Source: Genome Med. 2018 Oct 31;10:78. doi: 10.1186/s13073-018-0586-6 (PMC6208080; doi:10.1186/s13073-018-0586-6)
Supplement: Supplementary file 1 — Figure S1. Unweighted Unifrac distances between tumor and normal-associated microbiota in dMMR and pMMR. Figure S2. Venn diagram highlighting number of microbes that overlap between tumor and normal samples in relation to MMR status (dMMR = red font, pMMR = green font, red circles = tumor samples, blue circles =normal samples). Figure S3. Microbes significantly enriched in tumor as compared to normal samples (colon tissue and mucosa) in individuals with dMMR CRC: a) Bacteroides fragilis and b) Fusobacterium nucleatum. Figure S4. Differentially abundant OTUs between patient-matched tumor and normal samples in individuals with dMMR CRC from an independent cohort. Table S1. Table S1: List of PATRIC model IDs and associated sOTUs. Table S2. Microbes identified as differentially abundant in tumor as compared to normal samples (tissue and mucosa) from individuals with dMMR or pMMR CRC. Table S3. sOTUs enriched in tumor samples (colon tissue and mucosa) as compared to normal adjacent samples in individuals with dMMR CRC. Table S4. sOTUs enriched in tumor samples (colon tissue and mucosa) as compared to normal adjacent samples in individuals with pMMR CRC. Table S5. sOTUs enriched in the proximal or distal colon (colon tissue and mucosa) of individuals with dMMR CRC. Table S6. sOTUs enriched in the proximal or distal colon (colon tissue and mucosa) of individuals with pMMR CRC. Table S7. Differentially abundant microbes in individuals with dMMR CRC from an independent cohort. Table S8. Differentially abundant microbes in individuals with pMMR CRC from an independent cohort. (PDF 2790 kb) [file 13073_2018_586_MOESM1_ESM.pdf]

**Additional File**

**Figure S1:** Unweighted Unifrac distances between tumor and normal-associated microbiota in dMMR and pMMR.

**Figure S2**: Venn diagram highlighting number of microbes that overlap between tumor and normal samples in relation to MMR status (dMMR = red font, pMMR = green font, red circles = tumor samples, blue circles = normal samples). Only differentially abundant microbes with a corrected p-value < 0.05 were included in this diagram.

**Figure S3**: Microbes significantly enriched in tumor as compared to normal samples (colon tissue and mucosa) in individuals with dMMR CRC: a) *Bacteroides fragilis* and b) *Fusobacterium nucleatum*. Figures show relative abundances of each microbe microbe stratified by colon location (proximal or distal). Y-axis is square root transformed. For full results, please see Tables **S2**-**S6.**

**Figure S4**: Differentially abundant OTUs between patient–matched tumor and normal samples in individuals with dMMR CRC from an independent cohort. Y-axis indicates percent relative abundance of OTUs. Line color indicates directionality of change in microbial abundance: red = increased abundance relative to normal, blue = decreased abundance or no change compared to normal.

**Table S1**. Table S1: List of PATRIC model IDs and associated sOTUs.

**Table S2**. Microbes identified as differentially abundant in tumor as compared to normal samples (tissue and mucosa) from individuals with dMMR or pMMR CRC. Microbes are listed in order of significance from greatest to least. Microbes in bold font are enriched in both dMMR and pMMR samples. (Example: *Coprococcus comes ATCC 27758* is significantly enriched in dMMR tumor samples and pMMR normal samples.) (GLMM, all microbes listed have a Benjamini-Hochberg p-value<0.05)**Table S3**: sOTUs enriched in tumor samples (colon tissue and mucosa) as compared to normal–adjacent samples in individuals with dMMR CRC.

**Table S3**: sOTUs enriched in tumor samples (colon tissue and mucosa) as compared to normal adjacent samples in individuals with dMMR CRC.

**Table S4**: sOTUs enriched in tumor samples (colon tissue and mucosa) as compared to normal adjacent samples in individuals with pMMR CRC.

**Table S5**: sOTUs enriched in the proximal or distal colon (colon tissue and mucosa) of individuals with dMMR CRC.

**Table S6**: sOTUs enriched in the proximal or distal colon (colon tissue and mucosa) of individuals with pMMR CRC.

**Table S7**: Differentially abundant microbes in individuals with dMMR CRC from an independent cohort. Blue boxes highlight microbes enriched in tumor tissue samples as compared to normal–adjacent samples.

**Table S8**: Differentially abundant microbes in individuals with pMMR CRC from an independent cohort. Blue boxes highlight microbes enriched in tumor tissue samples as compared to normal–adjacent samples.

**Figure S1**

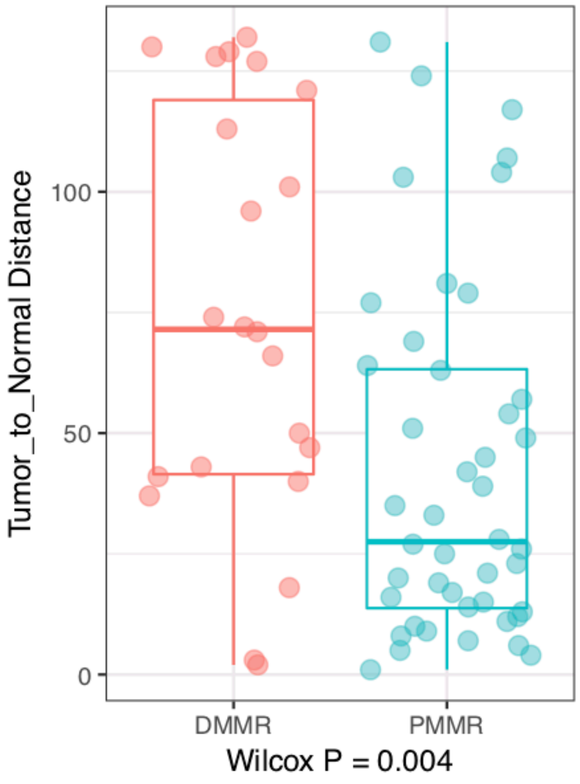


**Figure S2**

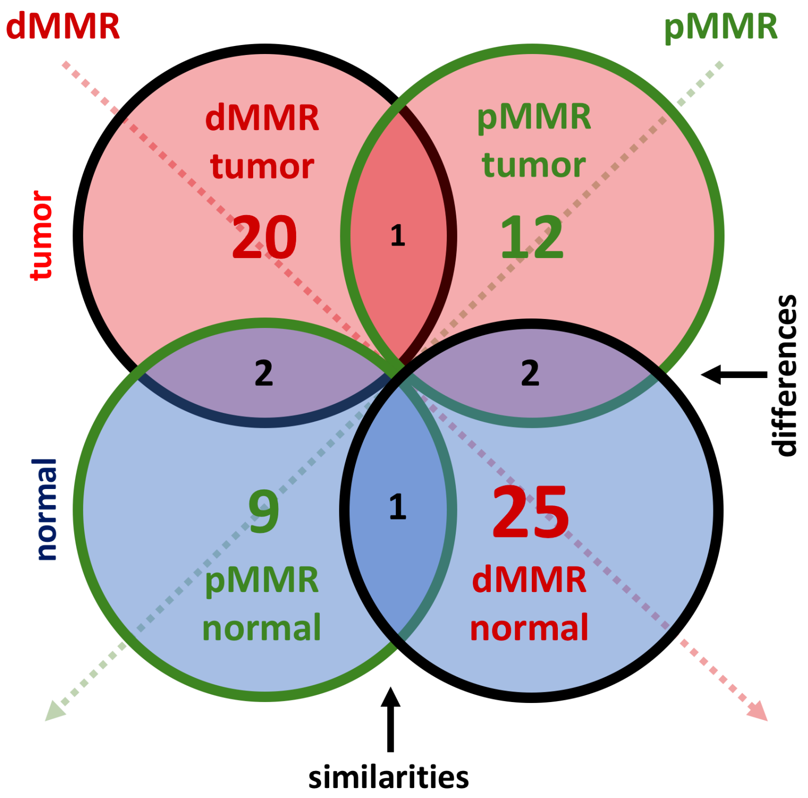

**Figure S3**

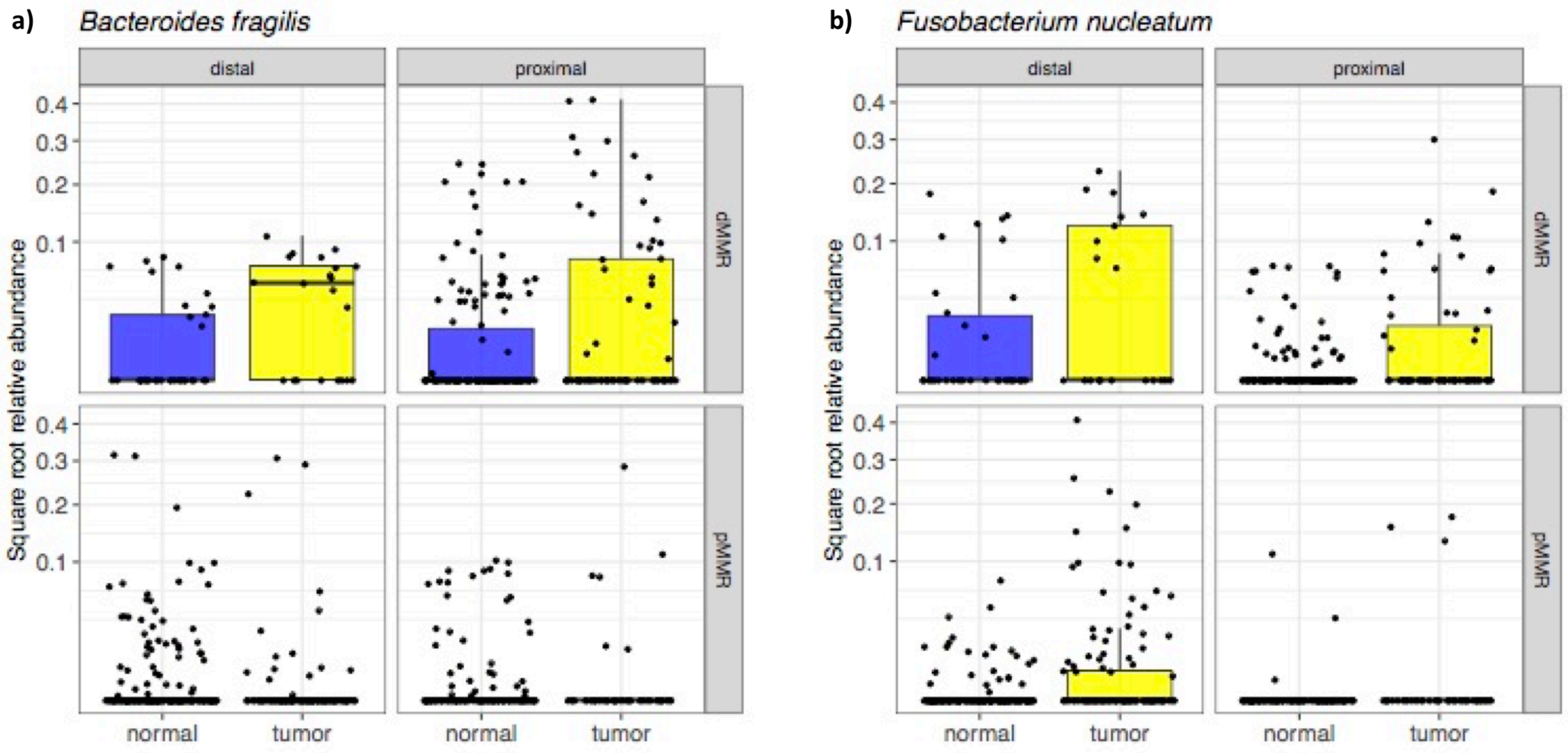
a)
Bacteroides fragilis
b)
Fusobacterium nucleatum
distal
proximal
dMMR
pMMR
Square root relative abundance
0.4
0.3
0.2
0.1
normal
tumor

**Figure S4**

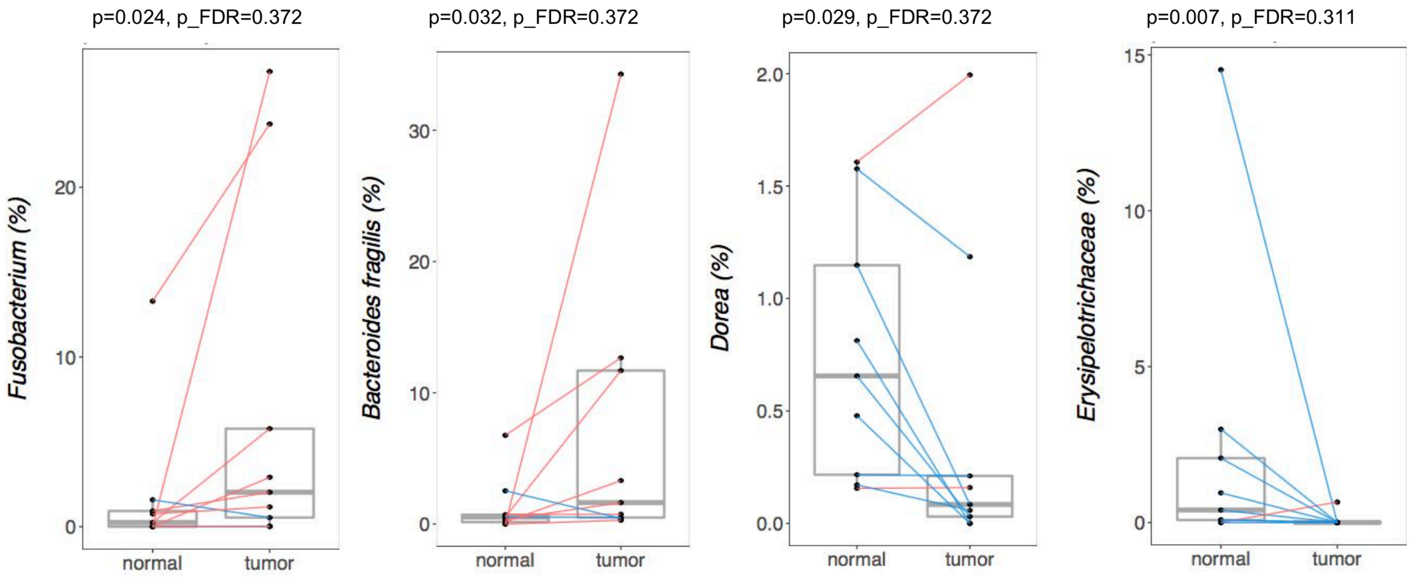
p=0.024, p_FDR=0.372
Fusobacterium (%)
0
10
20
normal
tumor
p=0.032, p_FDR=0.372
Bacteroides fragilis (%)
0
10
20
30
normal
tumor
p=0.029, p_FDR=0.372
Dorea (%)
0.0
0.5
1.0
1.5
2.0
normal
tumor
p=0.007, p_FDR=0.311
Erysipelotrichaceae (%)
0
5
10
15
normal
tumor

**Table S1**: List of PATRIC model IDs and associated sOTUs.

| model | associated sOTU | direction | influence score |
|---|---|---|---|
| 287.2537 | 9877e6b400d6b6ddd109860dd5833e9a6158a14e | Tumor | 45.97450752 |
| 1304.182 | 139bf28e70ca27abf9e9b4fd35182e3aa13554a4 | Normal | 38.92835722 |
| 1512.4 | 325635b4eea0f4ea47d598992cdd1fe791949d5f | Tumor | 30.12676086 |
| 28116.7 | 4a5d3ca1a784b0b59365e16d1d97f2f440751e15 | Tumor | 35.34356923 |
| 39488.4 | 3172c57e6f43b6dd00b72d6ba94b62cd4d386f07 | Tumor | 35.26014285 |
| 39490.3 | a9d3da348fab1229c75db0a1ff5b9a8d579bf3c5 | Normal | 7.816469481 |
| 88431.7 | b56b7e2dd2e753138a0a1d1e71f3056d12fed902 | Tumor | 13.35839489 |
| 214856.4 | eb6dc9f905c574e9ba6937eb007f0663e85ebf0f | Normal | 29.85772927 |
| 292800.4 | 15b516cadae4f56342986fc617b013ae81b5ce8a | Normal | 36.57608786 |
| 295405.11 | 8b87b344acea1c9d0217ea1774d20395a65c23d3 | Tumor | 31.76201761 |
| 360807.5 | b0e67ad37c689fd848f4ac9b923029d9c5b97b84 | Tumor | 5.04972306 |
| 370438.4 | 7a633438d89337bc55cd5c5fd04031625f5ec003 | Normal | 31.72251862 |
| 411460.6 | 0ee71c816b0045e83cc52804b39c593ef56f78ea | Normal | 29.45925917 |
| 411461.4 | fb4d2b05fa56f2555389e9e9f4c7ecfef185465c | Normal | 9.310550435 |
| 411462.6 | 04f784c78098595e60b39c56bc8831a1c246d8bd | Normal | 7.103736569 |
| 411462.6 | 071e09e58f93c64190d1facce0c7064963ca7ecb | Normal | 7.103736569 |
| 411483.3 | 7cb3cb4d27875a6896a122af34ee4e1e25c0658d | Normal | 34.23372632 |
| 411483.3 | 0c2b3a10e2af94b962ba7528f751204d55900582 | Normal | 34.23372632 |
| 435591.13 | 8707bac4e4749bf0b2c193cf8b359d5c0ac14986 | Tumor | 37.1416488 |
| 445972.6 | ab05bcb443299370fc72115db48e6b4157a86442 | Tumor | 34.65754847 |
| 450746.3 | 638bf3ad37e8c44d6598b006681ad9dbac9d6f3a | Normal | 5.80396739 |
| 457389.4 | 95a5fc04db20b1fee193023578b40f955999f970 | Normal | 34.48212012 |
| 457391.3 | b75db79d71e49907faca3540adab49d1720f69f7 | Normal | 33.80304752 |
| 469599.3 | c9a1c377ba9b93ac505d720a18ea2ed18f9e1fe6 | Tumor | 34.01896947 |
| 470146.3 | c7c054e78775fd54be9ea450fca5a2cc1945d3f8 | Tumor | 5.518796857 |
| 479437.5 | 5262929392a66cf2afc6a41089cb6dfc6d3e9160 | Tumor | 8.53215133 |
| 511145.12 | 84fe91323797b143ee23d1c9a0a119f74ac695f3 | Normal | 2.132791605 |
| 562982.3 | b3c6a6802f50753723c587db09737459a0ce4030 | Tumor | 38.82910147 |
| 596315.3 | 1f7d94002df355b116de0bddd570275227c9ef5b | Tumor | 10.78621211 |
| 657313.3 | 5098dddf48ee0ee369bfa4ae994436891b62bb42 | Normal | 0.624453092 |
| 657315.3 | 87c90d196bf14c4b33364104d9a1ba87f9ec7674 | Tumor | 10.73565791 |
| 657322.3 | cbe56d8d19aa6175f4664cbf2bc0532c68243477 | Normal | 7.089425206 |
| 663278.4 | fd1b56f2f52050a8575af68b53b5a691bea5db8c | Normal | 35.29485267 |
| 665956.3 | c996a0be142d3ef8c7bcf5b648ca29fdccb44d47 | Tumor | 13.72409367 |
| 991789.3 | 981787c105693e6d57f925437c828c382b0b5dcc | Normal | 27.6842406 |
| 997873.3 | 96e019fe5ef30fa9da8b0cf35c33928cac91f6e1 | Normal | 5.838643751 |
| 997886.3 | e3dabcc0d2ce7e6a9d0bedd684e9b9c924143ce5 | Normal | 41.41023915 |
| 999403.4 | f9f3f2e2c24d36b4d56f9b9edecc4f0817714b83 | Tumor | 37.28440165 |
| 1078089.3 | 92a9e6fd06b63cc48625058e4125b1a2f2cf3048 | Tumor | 38.72098734 |
| 1121098.3 | 714bb4b42c705998173a9e4009fa21cb94b32528 | Tumor | 28.8562891 |
| 1262706.3 | dd97ee7ed1ff7e48cf4248ea53130e398aadd0b0 | Normal | 33.70133074 |
| 1316586.3 | df4cfc980121095e2626e4d4f42c03fdd4c8428e | Tumor | 0.002482632 |
| 1414720.3 | 3de43bb82a62218309d99da68e342a6a97aab07f | Normal | 5.829584478 |
| 1504823.3 | a087ce6dca66a1ca488f78a8c14bb79845224cad | Normal | 12.63862858 |
| 1637506.3 | 9f1cd9232177f8118fc68906e04b28bcd454311b | Tumor | 7.588433686 |
| 1637509.3 | bc20137757c62ad131a83a32ef6141c46b9d6897 | Normal | 0.00189914 |
| 1658108.3 | 25fc8a0281b2e510ffcdce6d197757488c61e23b | Normal | 9.708804603 |
| 1737424.3 | c38a8f4266e22370fddb6dd2b031dd46c2ed486d | Normal | 37.96013856 |
| 1805476.3 | 85d5404085808330afd5d0bac27edee00bf513c5 | Normal | 8.732076254 |
| 1805476.3 | 96ea0ee772910b0790552195b224f430d863ba5a | Normal | 35.34293264 |

**Table S2**: Microbes identified as differentially abundant in tumor as compared to normal samples (tissue and mucosa) from individuals with dMMR or pMMR CRC. Microbes are listed in order of significance from greatest to least. Microbes in bold font are enriched in both dMMR and pMMR samples. (Example: *Coprococcus comes ATCC 27758* is significantly enriched in dMMR tumor samples and pMMR normal samples.) (GLMM, all microbes listed have a Benjamini-Hochberg p-value<0.05)

| dMMR Tumor | dMMR Normal |
|---|---|
| *Bacteroides fragilis YCH46*<br>*Fusobacterium periodonticum 2_1_31*<br>*Fusobacterium nucleatum CTI-5*<br>*Roseburia intestinalis M50/1*<br>*Bacteroides sp. HPS0048*<br>*Peptostreptococcus stomatis DSM 17678*<br>*Clostridium clostridioforme 90A1*<br>*[Clostridium] symbiosum*<br>*Pseudomonas aeruginosa strain NCTC10332*<br>***Coprococcus comes ATCC 27758***<br>*Eggerthella lenta DSM 2243*<br>*Roseburia inulinivorans*<br>***Bacteroides massiliensis B84634***<br>*Bacteroides vulgatus ATCC 8482*<br>*Parabacteroides distasonis ATCC 8503*<br>***Dorea longicatena***<br>*Lactobacillus sp. N15.MGS-260*<br>*Bacteroides ovatus strain ATCC 8483*<br>*Subdoligranulum sp. 4_3_54A2FAA*<br>*[Eubacterium] hallii* | *Bacteroides sp. 3_1_33FAA*<br>*Escherichia coli str. K-12 substr. MG1655*<br>*Ruminococcus torques ATCC 27756*<br>*Bacteroides vulgatus ATCC 8482*<br>*Bacteroides ovatus CL03T12C18*<br>*Faecalibacterium prausnitzii SL3/3*<br>***Faecalibacterium prausnitzii A2-165***<br>*Bacteroides sp. 3_1_13*<br>*Clostridium sp. JCC*<br>*Alistipes finegoldii*<br>*Lachnospiraceae bacterium mt14*<br>*bacterium LF-3*<br>***Blautia sp. Marseille-P2398***<br>*Coprobacillus sp. 8_1_38FAA*<br>*Ruminococcus torques L2-14*<br>*Eubacterium ramulus*<br>*Dorea longicatena DSM 13814*<br>*Streptococcus salivarius strain JF*<br>***Ethanoligenens harbinense YUAN-3***<br>*Bacteroides caccae CL03T12C61*<br>*Azospirillum sp. CAG:260*<br>*Dorea formicigenerans ATCC 27755*<br>*Pelotomaculum thermopropionicum SI*<br>*Acinetobacter sp. N54.MGS-139*<br>*[Clostridium] propionicum DSM 1682* |
| **pMMR Tumor** | **pMMR Normal** |
| *Prevotella copri DSM 18205*<br>*Ruminococcus sp. A254.MGS-108*<br>*Odoribacter splanchnicus DSM 220712*<br>*Campylobacter gracilis strain ATCC 33236*<br>*Bacteroides cellulosilyticus strain WH2*<br>*Bacteroides sp. 3_1_23*<br>*Firmicutes bacterium ASF500*<br>*Clostridium bolteae 90A9*<br>***Blautia sp. Marseille-P2398***<br>***Dorea longicatena***<br>***Faecalibacterium prausnitzii A2-165***<br>*Ruminococcus sp. A254.MGS-254* | ***Bacteroides massiliensis B84634***<br>***Ethanoligenens harbinense YUAN-3***<br>***Coprococcus comes ATCC 27758***<br>*Dialister invisus DSM 15470*<br>*Proteobacteria bacterium CAG:139*<br>*Clostridium cf. saccharolyticum K10*<br>*Fusicatenibacter saccharivorans*<br>*Holdemania filiformis DSM 12042*<br>*Burkholderia sp. K4410.MGS-135* |

**Table S3**: sOTUs enriched in tumor samples (colon tissue and mucosa) as compared to normal-adjacent samples in individuals with dMMR CRC.

| OTU | effect.size | pvalue | pvalueBH | direction | dMMR/pMMR | genome ID | influence | name |
|---|---|---|---|---|---|---|---|---|
| 8b87b344acea1c9d0217ea1774d20395a65c23d3 | 0.32487208 | 0 | 0 | Tumor | dMMR | 295405.11 | 927.0838 | Bacteroides fragilis YCH46 |
| b75db79d71e49907faca3540adab49d1720f69f7 | -0.4871055 | 0 | 0 | Normal | dMMR | 457391.3 | 1017.9764 | Bacteroides sp. 3_1_33FAA |
| c9a1c377ba9b93ac505d720a18ea2ed18f9e1fe6 | 0.91882064 | 0 | 0 | Tumor | dMMR | 469599.3 | 0 | Fusobacterium periodonticum 2_1_31 |
| df4cfc980121095e2626e4d4f42c03fdd4c8428e | 0.97009261 | 0 | 0 | Tumor | dMMR | 1316586.3 | 0 | Fusobacterium nucleatum CTI-5 |
| 84fe91323797b143ee23d1c9a0a119f74ac695f3 | -0.4259513 | 8.48E-179 | 1.27E-177 | Normal | dMMR | 511145.12 | 1452.8044 | Escherichia coli str. K-12 substr. MG1655 |
| 87c90d196bf14c4b33364104d9a1ba87f9ec7674 | 0.58496044 | 7.55E-126 | 9.43E-125 | Tumor | dMMR | 657315.3 | 1401.3243 | Roseburia intestinalis M50/1 |
| 0ee71c816b0045e83cc52804b39c593ef56f78ea | -0.6591072 | 2.04E-106 | 2.19E-105 | Normal | dMMR | 411460.6 | 1393.1467 | Ruminococcus torques ATCC 27756 |
| 5270d13a0a9b613c1ac12be16cf28f1839065233 | -0.4377876 | 1.45E-104 | 1.36E-103 | Normal | dMMR | 435590.9 | 1041.1283 | Bacteroides vulgatus ATCC 8482 |
| e3dabcc0d2ce7e6a9d0bedd684e9b9c924143ce5 | -0.6279138 | 2.41E-91 | 2.01E-90 | Normal | dMMR | 997886.3 | 1111.0023 | Bacteroides ovatus CL03T12C18 |
| 92a9e6fd06b63cc48625058e4125b1a2f2cf3048 | 0.59689464 | 4.99E-91 | 3.74E-90 | Tumor | dMMR | 1078089.3 | 1297.3163 | Bacteroides sp. HPS0048 |
| cbe56d8d19aa6175f4664cbf2bc0532c68243477 | -0.5931651 | 2.24E-86 | 1.53E-85 | Normal | dMMR | 657322.3 | 1395.953 | Faecalibacterium prausnitzii SL3/3 |
| 7cb3cb4d27875a6896a122af34ee4e1e25c0658d | -0.3841038 | 3.60E-85 | 2.25E-84 | Normal | dMMR | 411483.3 | 1615.3849 | Faecalibacterium prausnitzii A2-165 |
| 1f7d94002df355b116de0bddd570275227c9ef5b | 0.71386719 | 9.80E-82 | 5.65E-81 | Tumor | dMMR | 596315.3 | 156.4992 | Peptostreptococcus stomatis DSM 17678 |
| b3c6a6802f50753723c587db09737459a0ce4030 | 1.06029627 | 3.62E-79 | 1.94E-78 | Tumor | dMMR | 999403.4 | 1368.3447 | Clostridium clostridioforme 90A1 |
| 95a5fc04db20b1fee193023578b40f955999f970 | -0.4350349 | 2.49E-71 | 1.25E-70 | Normal | dMMR | 457389.4 | 1170.2812 | Bacteroides sp. 3_1_13 |
| 3de43bb82a62218309d99da68e342a6a97aab07f | -0.6675574 | 1.57E-69 | 7.36E-69 | Normal | dMMR | 1414720.3 | 906.6438 | Clostridium sp. JCC |
| 325635b4eea0f4ea47d598992cdd1fe791949d5f | 1.42677379 | 3.68E-56 | 1.62E-55 | Tumor | dMMR | 1512.4 | 1382.7717 | [Clostridium] symbiosum |
| eb6dc9f905c574e9ba6937eb007f0663e85ebf0f | -0.4261092 | 9.27E-46 | 3.86E-45 | Normal | dMMR | 214856.4 | 1028.712 | Alistipes finegoldii |
| 25fc8a0281b2e510ffcdce6d197757488c61e23b | -1.7122905 | 6.96E-42 | 2.75E-41 | Normal | dMMR | 1658108.3 | 916.2228 | Lachnospiraceae bacterium mt14 |
| a087ce6dca66a1ca488f78a8c14bb79845224cad | -1.1560302 | 3.74E-39 | 1.40E-38 | Normal | dMMR | 1504823.3 | 1243.0129 | bacterium LF-3 |
| 96ea0ee772910b0790552195b224f430d863ba5a | -0.4462376 | 1.16E-32 | 4.15E-32 | Normal | dMMR | 1805476.3 | 1202.5477 | Blautia sp. Marseille-P2398 |
| 0c2b3a10e2af94b962ba7528f751204d55900582 | -0.2694617 | 2.84E-28 | 9.67E-28 | Normal | dMMR | 411483.3 | 1615.3849 | Faecalibacterium prausnitzii A2-165 |
| f9f3f2e2c24d36b4d56f9b9edecc4f0817714b83 | 1.06069608 | 1.49E-27 | 4.85E-27 | Tumor | dMMR | 999403.4 | 1368.3447 | Clostridium clostridioforme 90A1 |
| 9877e6b400d6b6ddd109860dd5833e9a6158a14e | 0.7120549 | 3.89E-26 | 1.22E-25 | Tumor | dMMR | 287.2537 | 1732.7964 | Pseudomonas aeruginosa strain NCTC10332 |
| 638bf3ad37e8c44d6598b006681ad9dbac9d6f3a | -0.9666657 | 3.02E-22 | 9.05E-22 | Normal | dMMR | 450746.3 | 1363.6922 | Coprobacillus sp. 8_1_38FAA |
| 5098dddf48ee0ee369bfa4ae994436891b62bb42 | -0.4612898 | 5.40E-17 | 1.56E-16 | Normal | dMMR | 657313.3 | 1516.1855 | Ruminococcus torques L2-14 |
| 85d5404085808330afd5d0bac27edee00bf513c5 | -0.4564157 | 2.48E-16 | 6.89E-16 | Normal | dMMR | 1805476.3 | 1202.5477 | Blautia sp. Marseille-P2398 |
| c7c054e78775fd54be9ea450fca5a2cc1945d3f8 | 0.88602974 | 8.61E-16 | 2.31E-15 | Tumor | dMMR | 470146.3 | 1182.1518 | Coprococcus comes ATCC 27758 |
| a9d3da348fab1229c75db0a1ff5b9a8d579bf3c5 | -0.4264112 | 1.08E-14 | 2.79E-14 | Normal | dMMR | 39490.3 | 1407.3991 | Eubacterium ramulus |
| 071e09e58f93c64190d1facce0c7064963ca7ecb | -0.3480278 | 1.35E-14 | 3.38E-14 | Normal | dMMR | 411462.6 | 151.9632 | Dorea longicatena DSM 13814 |
| 139bf28e70ca27abf9e9b4fd35182e3aa13554a4 | -0.5072818 | 2.07E-12 | 4.85E-12 | Normal | dMMR | 1304.182 | 1395.7418 | Streptococcus salivarius strain JF |
| fd1b56f2f52050a8575af68b53b5a691bea5db8c | -0.5595702 | 4.41E-12 | 1.00E-11 | Normal | dMMR | 663278.4 | 1208.4086 | Ethanoligenens harbinense YUAN-3 |
| 5262929392a66cf2afc6a41089cb6dfc6d3e9160 | 1.06010194 | 5.84E-10 | 1.25E-09 | Tumor | dMMR | 479437.5 | 0 | Eggerthella lenta DSM 2243 |
| b0e67ad37c689fd848f4ac9b923029d9c5b97b84 | 0.41181276 | 1.39E-09 | 2.89E-09 | Tumor | dMMR | 360807.5 | 1193.0967 | Roseburia inulinivorans |
| 714bb4b42c705998173a9e4009fa21cb94b32528 | 0.3640049 | 2.02E-09 | 4.10E-09 | Tumor | dMMR | 1121098.3 | 795.1877 | Bacteroides massiliensis B84634 = Timone 84634 = DSM 17679 = JCM 13223 [PRJNA201686] |
| a882b133d4083f6fcc2ae50bd14dbe3d5446bb24 | 0.3309078 | 3.33E-08 | 6.58E-08 | Tumor | dMMR | 435590.9 | 1041.1283 | Bacteroides vulgatus ATCC 8482 |
| 8707bac4e4749bf0b2c193cf8b359d5c0ac14986 | 0.39743892 | 1.26E-07 | 2.43E-07 | Tumor | dMMR | 435591.13 | 915.4526 | Parabacteroides distasonis ATCC 8503 |
| 96e019fe5ef30fa9da8b0cf35c33928cac91f6e1 | -0.380227 | 5.51E-06 | 1.01E-05 | Normal | dMMR | 997873.3 | 996.4159 | Bacteroides caccae CL03T12C61 |
| b56b7e2dd2e753138a0a1d1e71f3056d12fed902 | 0.57075136 | 0.0008618 | 0.00143633 | Tumor | dMMR | 88431.7 | 1257.2534 | Dorea longicatena |
| dd97ee7ed1ff7e48cf4248ea53130e398aadd0b0 | -0.4085896 | 0.00126414 | 0.00197522 | Normal | dMMR | 1262706.3 | 155.0352 | Azospirillum sp. CAG:260 |
| 9f1cd9232177f8118fc68906e04b28bcd454311b | 0.51765698 | 0.00125275 | 0.00197522 | Tumor | dMMR | 1637506.3 | 155.1256 | Lactobacillus sp. N15.MGS-260 |
| fb4d2b05fa56f2555389e9e9f4c7ecfef185465c | -0.5372131 | 0.00150294 | 0.00230042 | Normal | dMMR | 411461.4 | 1407.3256 | Dorea formicigenerans ATCC 27755 |
| 7a633438d89337bc55cd5c5fd04031625f5ec003 | -0.5942097 | 0.00173382 | 0.00260073 | Normal | dMMR | 370438.4 | 1376.6485 | Pelotomaculum thermopropionicum SI |
| bc20137757c62ad131a83a32ef6141c46b9d6897 | -0.344278 | 0.00290228 | 0.00426806 | Normal | dMMR | 1637509.3 | 136.2402 | Acinetobacter sp. N54.MGS-139 |
| 4a5d3ca1a784b0b59365e16d1d97f2f440751e15 | 9.74443845 | 0.00865429 | 0.01248214 | Tumor | dMMR | 28116.7 | 1132.207 | Bacteroides ovatus strain ATCC 8483 |
| c996a0be142d3ef8c7bcf5b648ca29fdccb44d47 | 0.36683091 | 0.01032541 | 0.01461142 | Tumor | dMMR | 665956.3 | 1024.4306 | Subdoligranulum sp. 4_3_54A2FAA |
| 981787c105693e6d57f925437c828c382b0b5dcc | -0.2543321 | 0.0144519 | 0.01970714 | Normal | dMMR | 991789.3 | 1135.1437 | [Clostridium] propionicum DSM 1682 |
| 3172c57e6f43b6dd00b72d6ba94b62cd4d386f07 | 0.50177131 | 0.02391492 | 0.031467 | Tumor | dMMR | 39488.4 | 1194.9277 | [Eubacterium] hallii |
| 04f784c78098595e60b39c56bc8831a1c246d8bd | -1.7723933 | 0.0790503 | 0.10048767 | Normal | dMMR | 411462.6 | 151.9632 | Dorea longicatena DSM 13814 |
| ab05bcb443299370fc72115db48e6b4157a86442 | 0.40121918 | 0.10486613 | 0.13108266 | Tumor | dMMR | 445972.6 | 0 | Anaerotruncus colihominis DSM 17241 |
| c38a8f4266e22370fddb6dd2b031dd46c2ed486d | -0.264655 | 0.1180747 | 0.14517382 | Normal | dMMR | 1737424.3 | 1179.3562 | Blautia sp. GD8 |
| 15b516cadae4f56342986fc617b013ae81b5ce8a | -0.2961537 | 0.31878833 | 0.35685261 | Normal | dMMR | 292800.4 | 1431.4755 | Flavonifractor plautii |

**Table S4**: sOTUs enriched in tumor samples (colon tissue and mucosa) as compared to normal-adjacent samples in individuals with pMMR CRC.

| OTU | effect.size | pvalue | pvalueBH | direction | dMMR/pMMR | genome ID | Influence | name |
|---|---|---|---|---|---|---|---|---|
| e4bb49ce45f25c04d029c0f3c2a02ae1f780ed7e | -0.4050761 | 9.36E-47 | 4.73E-45 | Normal | pMMR | 1121098.3 | 833.960689 | Bacteroides massiliensis B84634 = Timone 84634 = DSM 17679 = JCM 13223 [PRJNA201686] |
| d81cca4cb1da96d4aac11b5e10da796b790f6f0d | 0.53859538 | 7.40E-37 | 1.49E-35 | Tumor | pMMR | 537011.5 | 827.7535 | Prevotella copri DSM 18205 |
| 630f4d47a9b05aa8f65f112233ab096262308e5b | -0.3243335 | 1.92E-30 | 3.24E-29 | Normal | pMMR | 1121098.7 | 733.813729 | Bacteroides massiliensis B84634 = Timone 84634 = DSM 17679 = JCM 13223 |
| d719fd0ae85ede0c04e8ec61275300fe178d3948 | 0.56566892 | 1.50E-27 | 2.16E-26 | Tumor | pMMR | 1637497.3 | 616.2185 | Ruminococcus sp. A254.MGS-108 |
| 8bac4e22d77ae1efc469563bab275f452e002119 | 0.3904693 | 5.68E-17 | 5.21E-16 | Tumor | pMMR | 709991.3 | 662.1316 | Odoribacter splanchnicus DSM 220712 |
| fa168b426edd535933f3a3828a6b828df49062a5 | 2.09593898 | 1.78E-13 | 1.29E-12 | Tumor | pMMR | 824.5 | 766.4971 | Campylobacter gracilis strain ATCC 33236 |
| a12bb28d688a2818753403165c417928c708839c | 0.2820261 | 1.04E-12 | 6.59E-12 | Tumor | pMMR | 246787.4 | 471.1197 | Bacteroides cellulosilyticus strain WH2 |
| 1c2b42abea5b6d81202bcc344ce570a43693931c | 0.27970625 | 1.76E-12 | 1.04E-11 | Tumor | pMMR | 457390.3 | 450.41 | Bacteroides sp. 3_1_23 |
| 0694fb7ab2fc435cd49b675a4a501d5d9daa2930 | -0.4289683 | 9.38E-10 | 4.12E-09 | Normal | pMMR | 663278.4 | 692.2454 | Ethanoligenens harbinense YUAN-3 |
| c7c054e78775fd54be9ea450fca5a2cc1945d3f8 | -0.451427 | 2.04E-08 | 8.57E-08 | Normal | pMMR | 470146.3 | 779.8438 | Coprococcus comes ATCC 27758 |
| 2b0c96c19e80e9f5a838843afb0b6c138d7172f7 | 0.28328637 | 3.73E-08 | 1.51E-07 | Tumor | pMMR | 1378168.3 | 1001.5082 | Firmicutes bacterium ASF500 |
| 9416469ebfb756921565fc178646cca9ca55d008 | -0.33991 | 7.77E-08 | 2.91E-07 | Normal | pMMR | 592028.3 | 657.798 | Dialister invisus DSM 15470 |
| b76e229e5444427d2ec5554d0665d25c8dace5ca | 0.32174448 | 1.40E-06 | 4.72E-06 | Tumor | pMMR | 997894.4 | 797.9159 | Clostridium bolteae 90A9 |
| 8b718f72b3c90530cc3cebbdab7e7d813658e679 | 0.59750509 | 7.94E-06 | 2.29E-05 | Tumor | pMMR | 1805476.3 | 675.950136 | Blautia sp. Marseille-P2398 |
| a7266616bf8e0fbf8eb72fe0988c33ef323183a1 | -0.3083376 | 1.10E-05 | 3.10E-05 | Normal | pMMR | 1262986.3 | 699.4664 | Proteobacteria bacterium CAG:139 |
| d7df7ac38235e7bfd8f7509a774a0023ff193d2a | -0.2518214 | 2.75E-05 | 7.50E-05 | Normal | pMMR | 717608.3 | 679.7108 | Clostridium cf. saccharolyticum K10 |
| 85f3c28e9e0bd6f89a28b03c277d3c0101634c45 | -0.287788 | 4.65E-05 | 0.00011462 | Normal | pMMR | 1150298.4 | 571.5477 | Fusicatenibacter saccharivorans |
| fd1b56f2f52050a8575af68b53b5a691bea5db8c | -0.3963119 | 1.04E-13 | 8.04E-13 | Normal | pMMR | 663278.4 | 692.2454 | Ethanoligenens harbinense YUAN-3 |
| b56b7e2dd2e753138a0a1d1e71f3056d12fed902 | 0.34825974 | 0.00121089 | 0.00246777 | Tumor | pMMR | 88431.7 | 821.9625 | Dorea longicatena |
| 26bf8c066146b41167a37935f3f358101696b6ee | -0.2768876 | 0.00122167 | 0.00246777 | Normal | pMMR | 545696.5 | 714.2144 | Holdemania filiformis DSM 12042 |
| aba2338cfa6a47bbec11e832e4034f127329942f | -22.191969 | 0.99675617 | 0.99999179 | Normal | pMMR | 717608.3 | 679.7108 | Clostridium cf. saccharolyticum K10 |
| 354517367a6dbbdf72794656076e945a8b727e27 | 0.85409107 | 0.0069283 | 0.01272287 | Tumor | pMMR | 411483.3 | 621.0295 | Faecalibacterium prausnitzii A2-165 |
| d0fcb4043b758d8c8b416265dce6280d2bc137a7 | -0.2757175 | 0.0087464 | 0.01577476 | Normal | pMMR | 1637500.3 | 734.2163 | Burkholderia sp. K4410.MGS-135 |
| b172803a089a26b95a72e38bd85201fa948cccdc | 1.62061816 | 0.00979644 | 0.0173586 | Tumor | pMMR | 1637499.3 | 951.214904 | Ruminococcus sp. A254.MGS-254 |
| 407411bafacade3dea8e72c2ec6880198037a6df | -1.645122 | 0.02941911 | 0.04952218 | Normal | pMMR | 445972.6 | 780.6051 | Anaerotruncus colihominis DSM 17241 |
| 4ce870e7ea166023ba98173c91b4451f7fdd0802 | -0.2915874 | 0.03593312 | 0.05949581 | Normal | pMMR | 428126.7 | 669.3468 | Clostridium spiroforme DSM 1552 |
| 9aabaca70ab3616bcc40b4e2e09a6d024049e7ee | -1.6820232 | 0.05708945 | 0.09152436 | Normal | pMMR | 1051074.3 | 489.8798 | Streptococcus thermophilus JIM 8232 |
| d6e7fa11f119f2b038716cba3ca721b8b39871bf | 0.32736017 | 0.06597265 | 0.10251135 | Tumor | pMMR | 1637504.3 | 694.1991 | Carnobacterium sp. N15.MGS-207 |
| 31feb7ea26c56019f324c5a0b5759129e162a7e1 | 0.37074939 | 0.07084033 | 0.1078422 | Tumor | pMMR | 40520.5 | 647.3191 | Blautia obeum |
| 3897e30a1778b4cbb87e053557cc96329d33fc01 | -0.705588 | 0.12163092 | 0.17549605 | Normal | pMMR | 1650661.3 | 621.865113 | Clostridium sp. GD3 |
| e9c75d4517ae2c00d0cc63923bece93188d46d6d | -1.103821 | 0.14520269 | 0.20655594 | Normal | pMMR | 457391.3 | 482.5388 | Bacteroides sp. 3_1_33FAA |
| 2b2000704aa739ea00a45a8bed5bdd42c8cafcb8 | 0.62362676 | 0.30456543 | 0.40475143 | Tumor | pMMR | 411463.4 | 662.936247 | Eubacterium ventriosum ATCC 27560 |
| 3172c57e6f43b6dd00b72d6ba94b62cd4d386f07 | 0.67857108 | 0.31880956 | 0.41281751 | Tumor | pMMR | 39488.4 | 743.1495 | Eubacterium hallii |
| 1bd214c8917453b714fe98affcff8616dee4d638 | 0.27669832 | 0.81501976 | 0.88512899 | Tumor | pMMR | 515620.4 | 719.9872 | Eubacterium eligens ATCC 27750 |
| df4cfc980121095e2626e4d4f42c03fdd4c8428e | 0.30515285 | 0.82946376 | 0.89123234 | Tumor | pMMR | 1316586.3 | 832.56 | Fusobacterium nucleatum CTI-5 |
| 83d8d058f7a1bc979398d8300b2b20084da9bfec | -8.5115812 | 0.91955476 | 0.9776319 | Normal | pMMR | 349741.6 | 643.5029 | Akkermansia muciniphila ATCC BAA-835 |
| 5674a6dcbc90f6ed5f456903ca7692c90d6c4d7b | -21.636361 | 0.99852384 | 0.99999179 | Normal | pMMR | 742725.3 | 629.4384 | Alistipes indistinctus YIT 12060 |

**Table S5:** sOTUs enriched in the proximal or distal colon (colon tissue and mucosa) of individuals with dMMR CRC.

| index | enriched in? | effect size | pvalue | pvalueBH | qvalue | taxonomy |
|---|---|---|---|---|---|---|
| 4a5d3ca1a784b0b59365e16d1d97f2f440751e15 | distal | 10.43 | 9.004E-03 | 3.554E-02 | 3.554E-02 | Bacteria;Bacteroidetes;Bacteroidia;Bacteroidales;Bacteroidaceae;Bacteroides;ovatus; |
| 638bf3ad37e8c44d6598b006681ad9dbac9d6f3a | distal | 2.09 | 1.932E-07 | 2.899E-06 | 2.899E-06 | Bacteria;Firmicutes;Erysipelotrichia;Erysipelotrichales;Erysipelotrichaceae;Erysipelotrichaceae_UCG-003;NA; |
| a882b133d4083f6fcc2ae50bd14dbe3d5446bb24 | distal | 1.91 | 5.581E-01 | 9.302E-01 | 9.302E-01 | Bacteria;Bacteroidetes;Bacteroidia;Bacteroidales;Bacteroidaceae;Bacteroides;dorei/sartorii/vulgatus; |
| 1f7d94002df355b116de0bddd570275227c9ef5b | distal | 1.73 | 1.849E-12 | 3.467E-11 | 3.467E-11 | Bacteria;Firmicutes;Clostridia;Clostridiales;Peptostreptococcaceae;Peptostreptococcus;stomatis; |
| ab05bcb443299370fc72115db48e6b4157a86442 | distal | 1.68 | 6.979E-01 | 9.999E-01 | 9.999E-01 | Bacteria;Firmicutes;Clostridia;Clostridiales;Ruminococcaceae;NA;NA; |
| 8c7e17334157e39923594420ee770f6ed1e5b608 | distal | 1.44 | 6.111E-01 | 9.353E-01 | 9.353E-01 | Bacteria;Firmicutes;Clostridia;Clostridiales;Lachnospiraceae;Tyzzerella;NA; |
| 8ebcc2adc430a757dbe9c835f2c0fe604ad07d14 | distal | 1.25 | 4.990E-02 | 1.439E-01 | 1.439E-01 | Bacteria;Firmicutes;Clostridia;Clostridiales;Lachnospiraceae;Dorea;formicigenerans; |
| eb6dc9f905c574e9ba6937eb007f0663e85ebf0f | distal | 1.17 | 4.934E-13 | 1.233E-11 | 1.233E-11 | Bacteria;Bacteroidetes;Bacteroidia;Bacteroidales;Rikenellaceae;Alistipes;finegoldii/onderdonkii; |
| 139bf28e70ca27abf9e9b4fd35182e3aa13554a4 | distal | 1.04 | 4.999E-01 | 8.719E-01 | 8.719E-01 | Bacteria;Firmicutes;Bacilli;Lactobacillales;Streptococcaceae;Streptococcus;NA; |
| 9f1cd9232177f8118fc68906e04b28bcd454311b | distal | 0.96 | 2.709E-03 | 1.355E-02 | 1.355E-02 | Bacteria;Firmicutes;Clostridia;Clostridiales;Ruminococcaceae;Ruminococcaceae_UCG-013;NA; |
| 3172c57e6f43b6dd00b72d6ba94b62cd4d386f07 | distal | 0.92 | 2.968E-06 | 3.180E-05 | 3.180E-05 | Bacteria;Firmicutes;Clostridia;Clostridiales;Lachnospiraceae;NA;NA; |
| 5098dddf48ee0ee369bfa4ae994436891b62bb42 | distal | 0.82 | 4.218E-02 | 1.318E-01 | 1.318E-01 | Bacteria;Firmicutes;Clostridia;Clostridiales;Lachnospiraceae;NA;NA; |
| 249bed28190c2a1fa1b808457c4bb15912e4722d | distal | 0.66 | 1.527E-03 | 8.812E-03 | 8.812E-03 | Bacteria;Firmicutes;Clostridia;Clostridiales;Lachnospiraceae;NA;NA; |
| 15b516cadae4f56342986fc617b013ae81b5ce8a | distal | 0.59 | 1.856E-01 | 4.429E-01 | 4.429E-01 | Bacteria;Firmicutes;Clostridia;Clostridiales;Ruminococcaceae;Flavonifractor;plautii; |
| c38a8f4266e22370fddb6dd2b031dd46c2ed486d | distal | 0.53 | 1.623E-01 | 4.222E-01 | 4.222E-01 | Bacteria;Firmicutes;Clostridia;Clostridiales;Lachnospiraceae;Blautia;massiliensis; |
| c7c054e78775fd54be9ea450fca5a2cc1945d3f8 | distal | 0.50 | 2.490E-01 | 5.501E-01 | 5.501E-01 | Bacteria;Firmicutes;Clostridia;Clostridiales;Lachnospiraceae;Tyzzerella_4;genus_check/comes; |
| 88280c5eef5b050e56011169b897086055a3e4a7 | distal | 0.42 | 3.458E-01 | 6.825E-01 | 6.825E-01 | Bacteria;Actinobacteria;Coriobacteriia;Coriobacteriales;Eggerthellaceae;Eggerthella;lenta; |
| cbe56d8d19aa6175f4664cbf2bc0532c68243477 | distal | 0.41 | 3.221E-03 | 1.510E-02 | 1.510E-02 | Bacteria;Firmicutes;Clostridia;Clostridiales;Ruminococcaceae;Faecalibacterium;prausnitzii; |
| 9e481a0c094b8dc9690018e5bcc929dfb8c43189 | distal | 0.29 | 4.867E-01 | 8.692E-01 | 8.692E-01 | Bacteria;Firmicutes;Erysipelotrichia;Erysipelotrichales;Erysipelotrichaceae;Solobacterium;moorei; |
| 04f784c78098595e60b39c56bc8831a1c246d8bd | distal | 0.26 | 8.508E-01 | 9.999E-01 | 9.999E-01 | Bacteria;Firmicutes;Clostridia;Clostridiales;Lachnospiraceae;Dorea;genus_check/longicatena/torques; |
| 5262929392a66cf2afc6a41089cb6dfc6d3e9160 | distal | 0.24 | 5.335E-01 | 9.094E-01 | 9.094E-01 | Bacteria;Actinobacteria;Coriobacteriia;Coriobacteriales;Eggerthellaceae;Eggerthella;lenta; |
| 87c90d196bf14c4b33364104d9a1ba87f9ec7674 | distal | 0.22 | 2.661E-03 | 1.355E-02 | 1.355E-02 | Bacteria;Firmicutes;Clostridia;Clostridiales;Lachnospiraceae;Roseburia;intestinalis; |
| a6aa29c3b174f2e3e608d8bd34b7b4140345d7df | distal | 0.13 | 5.897E-01 | 9.353E-01 | 9.353E-01 | Bacteria;Firmicutes;Clostridia;Clostridiales;Lachnospiraceae;Blautia;faecis; |
| a087ce6dca66a1ca488f78a8c14bb79845224cad | distal | 0.11 | 8.869E-01 | 9.999E-01 | 9.999E-01 | Bacteria;Firmicutes;Erysipelotrichia;Erysipelotrichales;Erysipelotrichaceae;Erysipelotrichaceae_UCG-003;NA; |
| a9d3da348fab1229c75db0a1ff5b9a8d579bf3c5 | distal | 0.08 | 6.078E-01 | 9.353E-01 | 9.353E-01 | Bacteria;Firmicutes;Clostridia;Clostridiales;Lachnospiraceae;Agathobacter;NA; |
| 5472c79c6d7f84d65454bdc880e450071a430607 | distal | 0.06 | 7.298E-01 | 9.999E-01 | 9.999E-01 | Bacteria;Firmicutes;Clostridia;Clostridiales;Ruminococcaceae;Faecalibacterium;cf./prausnitzii; |
| df4cfc980121095e2626e4d4f42c03fdd4c8428e | proximal | -0.02 | 9.774E-01 | 9.999E-01 | 9.999E-01 | Bacteria;Fusobacteria;Fusobacteriia;Fusobacteriales;Fusobacteriaceae;Fusobacterium;nucleatum; |
| b0e2c85ee364dc831f99bca252f35e2f06afd5f6 | proximal | -0.02 | 9.002E-01 | 9.999E-01 | 9.999E-01 | Bacteria;Firmicutes;Clostridia;Clostridiales;Ruminococcaceae;Ruminococcaceae_UCG-002;NA; |
| 96ea0ee772910b0790552195b224f430d863ba5a | proximal | -0.03 | 8.264E-01 | 9.999E-01 | 9.999E-01 | Bacteria;Firmicutes;Clostridia;Clostridiales;Lachnospiraceae;Blautia;luti/obeum; |
| 3de43bb82a62218309d99da68e342a6a97aab07f | proximal | -0.11 | 1.890E-01 | 4.429E-01 | 4.429E-01 | Bacteria;Firmicutes;Clostridia;Clostridiales;Clostridiaceae_1;Clostridium_sensu_stricto_1;celatum/disporicum/saudiense; |
| 0c2b3a10e2af94b962ba7528f751204d55900582 | proximal | -0.15 | 2.494E-01 | 5.501E-01 | 5.501E-01 | Bacteria;Firmicutes;Clostridia;Clostridiales;Ruminococcaceae;Faecalibacterium;prausnitzii; |
| 1910ca1b089d8231b2e67f8454cdbd5c020c3144 | proximal | -0.20 | 6.014E-01 | 9.353E-01 | 9.353E-01 | Bacteria;Firmicutes;Erysipelotrichia;Erysipelotrichales;Erysipelotrichaceae;Erysipelatoclostridium;ramosum; |
| a7266616bf8e0fbf8eb72fe0988c33ef323183a1 | proximal | -0.21 | 4.632E-01 | 8.473E-01 | 8.473E-01 | Bacteria;Proteobacteria;Gammaproteobacteria;Betaproteobacteriales;Burkholderiaceae;Parasutterella;NA; |
| 071e09e58f93c64190d1facce0c7064963ca7ecb | proximal | -0.22 | 1.604E-01 | 4.222E-01 | 4.222E-01 | Bacteria;Firmicutes;Clostridia;Clostridiales;Lachnospiraceae;Dorea;longicatena; |
| ef17e279b408157cedec42487d1caa56d911ace4 | proximal | -0.32 | 4.870E-05 | 3.653E-04 | 3.653E-04 | Bacteria;Firmicutes;Clostridia;Clostridiales;Lachnospiraceae;Agathobacter;NA; |
| 7cb3cb4d27875a6896a122af34ee4e1e25c0658d | proximal | -0.35 | 2.743E-05 | 2.286E-04 | 2.286E-04 | Bacteria;Firmicutes;Clostridia;Clostridiales;Ruminococcaceae;Faecalibacterium;CM04-06; |
| 647fb0235ac545a07e5ed6c880b2318e6f45df29 | proximal | -0.37 | 1.028E-04 | 7.007E-04 | 7.007E-04 | Bacteria;Bacteroidetes;Bacteroidia;Bacteroidales;Bacteroidaceae;Bacteroides;faecis/thetaiotaomicron; |
| b75db79d71e49907faca3540adab49d1720f69f7 | proximal | -0.40 | 4.621E-21 | 1.733E-19 | 1.733E-19 | Bacteria;Bacteroidetes;Bacteroidia;Bacteroidales;Bacteroidaceae;Bacteroides;dorei/vulgatus; |
| e3dabcc0d2ce7e6a9d0bedd684e9b9c924143ce5 | proximal | -0.42 | 1.145E-02 | 4.293E-02 | 4.293E-02 | Bacteria;Bacteroidetes;Bacteroidia;Bacteroidales;Bacteroidaceae;Bacteroides;fragilis/ovatus; |
| c996a0be142d3ef8c7bcf5b648ca29fdccb44d47 | proximal | -0.49 | 3.442E-01 | 6.825E-01 | 6.825E-01 | Bacteria;Firmicutes;Clostridia;Clostridiales;Ruminococcaceae;UBA1819;NA; |
| 7a633438d89337bc55cd5c5fd04031625f5ec003 | proximal | -0.51 | 7.917E-01 | 9.999E-01 | 9.999E-01 | Bacteria;Firmicutes;Clostridia;Clostridiales;Christensenellaceae;Christensenellaceae_R-7_group;NA; |
| f9f3f2e2c24d36b4d56f9b9edecc4f0817714b83 | proximal | -0.58 | 3.659E-01 | 7.037E-01 | 7.037E-01 | Bacteria;Firmicutes;Clostridia;Clostridiales;Lachnospiraceae;Lachnoclostridium;urinimassiliense; |
| 25fc8a0281b2e510ffcdce6d197757488c61e23b | proximal | -0.71 | 1.633E-01 | 4.222E-01 | 4.222E-01 | Bacteria;Firmicutes;Clostridia;Clostridiales;Lachnospiraceae;Epulopiscium;NA; |
| 1e2a1be1c43888849af6f4d6fea70dc701655379 | proximal | -0.75 | 6.557E-03 | 2.732E-02 | 2.732E-02 | Bacteria;Firmicutes;Clostridia;Clostridiales;Peptostreptococcaceae;Intestinibacter;bartlettii; |
| 0ee71c816b0045e83cc52804b39c593ef56f78ea | proximal | -0.79 | 3.626E-06 | 3.399E-05 | 3.399E-05 | Bacteria;Firmicutes;Clostridia;Clostridiales;Lachnospiraceae;NA;NA; |
| 6c63c7c4b65e18ecb6da353513b0dc9620820947 | proximal | -0.89 | 7.287E-04 | 4.554E-03 | 4.554E-03 | Bacteria;Firmicutes;Clostridia;Clostridiales;Ruminococcaceae;Oscillibacter;NA; |
| 5270d13a0a9b613c1ac12be16cf28f1839065233 | proximal | -0.93 | 7.728E-01 | 9.999E-01 | 9.999E-01 | Bacteria;Bacteroidetes;Bacteroidia;Bacteroidales;Bacteroidaceae;Bacteroides;dorei/vulgatus; |
| 1a5db7cbf3e568848481d107f6f2da7e423425c1 | proximal | -0.95 | 6.356E-01 | 9.534E-01 | 9.534E-01 | Bacteria;Bacteroidetes;Bacteroidia;Bacteroidales;Marinifilaceae;Odoribacter;splanchnicus; |
| fb4d2b05fa56f2555389e9e9f4c7ecfef185465c | proximal | -0.97 | 7.437E-01 | 9.999E-01 | 9.999E-01 | Bacteria;Firmicutes;Clostridia;Clostridiales;Lachnospiraceae;Dorea;genus_check/formicigenerans/torques; |
| b0e67ad37c689fd848f4ac9b923029d9c5b97b84 | proximal | -1.19 | 3.311E-01 | 6.825E-01 | 6.825E-01 | Bacteria;Firmicutes;Clostridia;Clostridiales;Lachnospiraceae;Roseburia;inulinivorans; |
| 8b87b344acea1c9d0217ea1774d20395a65c23d3 | proximal | -1.26 | 3.145E-02 | 1.072E-01 | 1.072E-01 | Bacteria;Bacteroidetes;Bacteroidia;Bacteroidales;Bacteroidaceae;Bacteroides;dorei/fragilis; |
| afb8924308524d3194cd3aefe538773646df563d | proximal | -1.60 | 4.190E-01 | 7.855E-01 | 7.855E-01 | Bacteria;Firmicutes;Clostridia;Clostridiales;Ruminococcaceae;Ruminococcaceae_UCG-004;NA; |
| 3897e30a1778b4cbb87e053557cc96329d33fc01 | proximal | -2.41 | 1.698E-01 | 4.245E-01 | 4.245E-01 | Bacteria;Firmicutes;Clostridia;Clostridiales;Ruminococcaceae;Ruminiclostridium_9;NA; |
| 64de730cea6eb29df43c1257bcebb75eece2daea | proximal | -2.74 | 3.290E-02 | 1.073E-01 | 1.073E-01 | Bacteria;Bacteroidetes;Bacteroidia;Bacteroidales;Bacteroidaceae;Bacteroides;fragilis/xylanisolvens; |
| 9877e6b400d6b6ddd109860dd5833e9a6158a14e | proximal | -2.81 | 4.484E-02 | 1.345E-01 | 1.345E-01 | Bacteria;Proteobacteria;Gammaproteobacteria;Pseudomonadales;Pseudomonadaceae;Pseudomonas;aeruginosa/alcaligenes/citronellolis/delhiensis/denitrificans/fluorescens/guezennei/knackmussii/mendocina/mosselii/nitroreducens/otitidis/protegens/pseudoalcaligenes/putida/resinovorans/stutzeri/tropicalis; |
| 2b0c96c19e80e9f5a838843afb0b6c138d7172f7 | proximal | -3.01 | 2.598E-01 | 5.567E-01 | 5.567E-01 | Bacteria;Firmicutes;Clostridia;Clostridiales;Ruminococcaceae;Ruminiclostridium_9;NA; |
| 95a5fc04db20b1fee193023578b40f955999f970 | proximal | -3.44 | 1.617E-168 | 1.213E-166 | 1.213E-166 | Bacteria;Bacteroidetes;Bacteroidia;Bacteroidales;Bacteroidaceae;Bacteroides;acidifaciens/caccae/caecimuris/dorei/faecichinchillae/faecis/fragilis/graminisolvens/neonati/ovatus/reticulotermitis/salyersiae/thetaiotaomicron/xylanisolvens; |
| edce63d18074b04557d5b57df0988d78e4a4ee6f | proximal | -3.45 | 2.476E-07 | 3.095E-06 | 3.095E-06 | Bacteria;Firmicutes;Clostridia;Clostridiales;Lachnospiraceae;Lachnospiraceae_NK4A136_group;NA; |
| 84fe91323797b143ee23d1c9a0a119f74ac695f3 | proximal | -4.08 | 2.020E-02 | 7.215E-02 | 7.215E-02 | Bacteria;Proteobacteria;Gammaproteobacteria;Enterobacteriales;Enterobacteriaceae;Escherichia/Shigella;albertii/boydii/coli/dysenteriae/fergusonii/flexneri/sonnei/vulneris; |
| 85d5404085808330afd5d0bac27edee00bf513c5 | proximal | -4.88 | 5.627E-03 | 2.482E-02 | 2.482E-02 | Bacteria;Firmicutes;Clostridia;Clostridiales;Lachnospiraceae;Blautia;luti/obeum/wexlerae; |
| d97ae445fd4a641bd2c19b3f253967d251a8579b | proximal | -14.55 | 9.980E-01 | 9.999E-01 | 9.999E-01 | Bacteria;Firmicutes;Clostridia;Clostridiales;Family_XI;Anaerococcus;vaginalis; |
| dd97ee7ed1ff7e48cf4248ea53130e398aadd0b0 | proximal | -15.13 | 9.986E-01 | 9.999E-01 | 9.999E-01 | Bacteria;Proteobacteria;Alphaproteobacteria;Rhodospirillales;NA;NA;NA; |
| 8707bac4e4749bf0b2c193cf8b359d5c0ac14986 | proximal | -15.65 | 9.980E-01 | 9.999E-01 | 9.999E-01 | Bacteria;Bacteroidetes;Bacteroidia;Bacteroidales;Tannerellaceae;Parabacteroides;distasonis; |
| 981787c105693e6d57f925437c828c382b0b5dcc | proximal | -16.58 | 9.980E-01 | 9.999E-01 | 9.999E-01 | Bacteria;Firmicutes;Clostridia;Clostridiales;Lachnospiraceae;Tyzzerella;NA; |
| fd1b56f2f52050a8575af68b53b5a691bea5db8c | proximal | -17.56 | 9.975E-01 | 9.999E-01 | 9.999E-01 | Bacteria;Firmicutes;Clostridia;Clostridiales;Ruminococcaceae;Negativibacillus;NA; |
| 87e27f8ad1f4b012359a25d7918bb7478578b58d | proximal | -19.40 | 9.973E-01 | 9.999E-01 | 9.999E-01 | Bacteria;Firmicutes;Clostridia;Clostridiales;Lachnospiraceae;NA;NA; |
| b3c6a6802f50753723c587db09737459a0ce4030 | proximal | -20.60 | 9.966E-01 | 9.999E-01 | 9.999E-01 | Bacteria;Firmicutes;Bacilli;Bacillales;Family_XI;Gemella;morbillorum; |
| 92a9e6fd06b63cc48625058e4125b1a2f2cf3048 | proximal | -20.86 | 9.915E-01 | 9.999E-01 | 9.999E-01 | Bacteria;Bacteroidetes;Bacteroidia;Bacteroidales;Bacteroidaceae;Bacteroides;nordii; |
| 325635b4eea0f4ea47d598992cdd1fe791949d5f | proximal | -20.99 | 9.967E-01 | 9.999E-01 | 9.999E-01 | Bacteria;Firmicutes;Clostridia;Clostridiales;Lachnospiraceae;Lachnoclostridium;NA; |
| b56b7e2dd2e753138a0a1d1e71f3056d12fed902 | proximal | -21.95 | 9.984E-01 | 9.999E-01 | 9.999E-01 | Bacteria;Firmicutes;Clostridia;Clostridiales;Lachnospiraceae;NA;NA; |
| 67db4f7e9b0c521c1a7a813a1b0874baa173161f | proximal | -22.25 | 9.980E-01 | 9.999E-01 | 9.999E-01 | Bacteria;Firmicutes;Clostridia;Clostridiales;Peptostreptococcaceae;Intestinibacter;genus_check/bartlettii/ilealis/timonensis; |
| bc20137757c62ad131a83a32ef6141c46b9d6897 | proximal | -22.41 | 9.975E-01 | 9.999E-01 | 9.999E-01 | Bacteria;Firmicutes;Clostridia;Clostridiales;Lachnospiraceae;Lachnospiraceae_UCG-004;NA; |
| 96e019fe5ef30fa9da8b0cf35c33928cac91f6e1 | proximal | -23.27 | 9.974E-01 | 9.999E-01 | 9.999E-01 | Bacteria;Bacteroidetes;Bacteroidia;Bacteroidales;Bacteroidaceae;Bacteroides;caccae; |
| 714bb4b42c705998173a9e4009fa21cb94b32528 | proximal | -24.17 | 9.981E-01 | 9.999E-01 | 9.999E-01 | Bacteria;Bacteroidetes;Bacteroidia;Bacteroidales;Bacteroidaceae;Bacteroides;massiliensis; |
| c9a1c377ba9b93ac505d720a18ea2ed18f9e1fe6 | proximal | -31.01 | 9.999E-01 | 9.999E-01 | 9.999E-01 | Bacteria;Fusobacteria;Fusobacteriia;Fusobacteriales;Fusobacteriaceae;Fusobacterium;nucleatum/periodonticum; |

**Table S6**: sOTUs enriched in the proximal or distal colon (colon tissue and mucosa) of individuals with pMMR CRC.

| index | enriched in? | effect size | pvalue | pvalueBH | qvalue | taxonomy |
|---|---|---|---|---|---|---|
| df47ea78f6179ac451d12468f5085395ba4d8240 | distal | 25.42 | 0.99558244 | 0.99993574 | 0.44440924 | Bacteria;Bacteroidetes;Bacteroidia;Bacteroidales;Bacteroidaceae;Bacteroides;stercoris; |
| 1bd214c8917453b714fe98affcff8616dee4d638 | distal | 2.19 | 1.46E-05 | 7.39E-05 | 3.29E-05 | Bacteria;Firmicutes;Clostridia;Clostridiales;Lachnospiraceae;Lachnospira;genus_check/catus/eligens/pectinophilus/pectinoschiza; |
| 7560aeb50e1f8adfb7c74235c38ae9c9a33d8378 | distal | 1.73 | 0.62825714 | 0.7465173 | 0.33178051 | Bacteria;Bacteroidetes;Bacteroidia;Bacteroidales;Prevotellaceae;Prevotella_7;NA; |
| 85467fd860872ebd158f2f3d9bb38ab46146c531 | distal | 1.49 | 0.69720004 | 0.78409559 | 0.34848172 | Bacteria;Bacteroidetes;Bacteroidia;Bacteroidales;Barnesiellaceae;Barnesiella;intestinihominis; |
| b172803a089a26b95a72e38bd85201fa948cccdc | distal | 1.08 | 0.00643745 | 0.02167275 | 0.00963219 | Bacteria;Firmicutes;Clostridia;Clostridiales;Ruminococcaceae;NA;NA; |
| d2365539ad0544895f24005f922027645ef7d892 | distal | 0.99 | 0.03578512 | 0.08394285 | 0.03730738 | Bacteria;Firmicutes;Clostridia;Clostridiales;Lachnospiraceae;Roseburia;hominis; |
| 31feb7ea26c56019f324c5a0b5759129e162a7e1 | distal | 0.86 | 6.62E-07 | 4.18E-06 | 1.86E-06 | Bacteria;Firmicutes;Clostridia;Clostridiales;Lachnospiraceae;GCA-900066575;NA; |
| 41b14722c5a50fc51fa4f53d380a2877dc322e3a | distal | 0.80 | 0.0720636 | 0.13996969 | 0.06220782 | Bacteria;Firmicutes;Clostridia;Clostridiales;Ruminococcaceae;Oscillibacter;NA; |
| 83d8d058f7a1bc979398d8300b2b20084da9bfec | distal | 0.77 | 0.65283205 | 0.76001379 | 0.33777885 | Bacteria;Verrucomicrobia;Verrucomicrobiae;Verrucomicrobiales;Akkermansiaceae;Akkermansia;muciniphila; |
| fd721a257132cf7b8ae457f7892e169593483d6f | distal | 0.77 | 0.22589621 | 0.38025862 | 0.1690013 | Bacteria;Firmicutes;Clostridia;Clostridiales;Lachnospiraceae;Blautia;genus_check/massiliensis/saccharivorans; |
| bfa061a1d7abda128775ebfbbbda5f7ab193bc56 | distal | 0.71 | 0.23917109 | 0.39076461 | 0.17367056 | Bacteria;Bacteroidetes;Bacteroidia;Bacteroidales;Bacteroidaceae;Bacteroides;fragilis; |
| 3c859dbc46f4dea88830a478c2141721a436bdcc | distal | 0.65 | 5.54E-09 | 4.30E-08 | 1.91E-08 | Bacteria;Firmicutes;Clostridia;Clostridiales;Ruminococcaceae;Ruminococcaceae_UCG-002;NA; |
| 5674a6dcbc90f6ed5f456903ca7692c90d6c4d7b | distal | 0.64 | 0.30566033 | 0.44741585 | 0.19884851 | Bacteria;Bacteroidetes;Bacteroidia;Bacteroidales;Rikenellaceae;Alistipes;indistinctus; |
| c1edcf05d753b1b78a9e18cf4140ae23908835c1 | distal | 0.57 | 4.87E-05 | 0.00021404 | 9.51E-05 | Bacteria;Bacteroidetes;Bacteroidia;Bacteroidales;Bacteroidaceae;Bacteroides;thetaiotaomicron; |
| b0e2c85ee364dc831f99bca252f35e2f06afd5f6 | distal | 0.55 | 1.06E-05 | 5.65E-05 | 2.51E-05 | Bacteria;Firmicutes;Clostridia;Clostridiales;Ruminococcaceae;Ruminococcaceae_UCG-002;NA; |
| 8b718f72b3c90530cc3cebbdab7e7d813658e679 | distal | 0.55 | 0.00301611 | 0.0105044 | 0.00466855 | Bacteria;Firmicutes;Clostridia;Clostridiales;Lachnospiraceae;Blautia;wexlerae; |
| e3a547cc9e32ef7c319cd82c47b3862462234c0f | distal | 0.53 | 2.14E-17 | 2.40E-16 | 1.07E-16 | Bacteria;Proteobacteria;Gammaproteobacteria;Betaproteobacteriales;Burkholderiaceae;Sutterella;wadsworthensis; |
| 4ce870e7ea166023ba98173c91b4451f7fdd0802 | distal | 0.48 | 0.00738089 | 0.02329593 | 0.01035359 | Bacteria;Firmicutes;Erysipelotrichia;Erysipelotrichales;Erysipelotrichaceae;Erysipelatoclostridium;NA; |
| d81cca4cb1da96d4aac11b5e10da796b790f6f0d | distal | 0.42 | 1.22E-19 | 2.05E-18 | 9.13E-19 | Bacteria;Bacteroidetes;Bacteroidia;Bacteroidales;Prevotellaceae;Prevotella_9;NA; |
| d719fd0ae85ede0c04e8ec61275300fe178d3948 | distal | 0.38 | 1.66E-07 | 1.12E-06 | 4.97E-07 | Bacteria;Firmicutes;Clostridia;Clostridiales;Ruminococcaceae;Ruminiclostridium_5;NA; |
| 8f83249ba2ebecfe9a9e354a6cc159d752107b51 | distal | 0.38 | 1.19E-63 | 5.99E-62 | 2.66E-62 | Bacteria;Bacteroidetes;Bacteroidia;Bacteroidales;Tannerellaceae;Parabacteroides;distasonis; |
| 354517367a6dbbdf72794656076e945a8b727e27 | distal | 0.37 | 0.30265387 | 0.44741585 | 0.19884851 | Bacteria;Firmicutes;Clostridia;Clostridiales;Ruminococcaceae;Faecalibacterium;AF52-21/prausnitzii; |
| 1c2b42abea5b6d81202bcc344ce570a43693931c | distal | 0.36 | 2.11E-22 | 5.33E-21 | 2.37E-21 | Bacteria;Bacteroidetes;Bacteroidia;Bacteroidales;Bacteroidaceae;Bacteroides;ovatus; |
| 4437afcc0fee950906aabdea95de3216b52eb175 | distal | 0.36 | 3.65E-106 | 3.68E-104 | 1.64E-104 | Bacteria;Bacteroidetes;Bacteroidia;Bacteroidales;Bacteroidaceae;Bacteroides;caccae; |
| 85f3c28e9e0bd6f89a28b03c277d3c0101634c45 | distal | 0.31 | 8.63E-06 | 4.84E-05 | 2.15E-05 | Bacteria;Firmicutes;Clostridia;Clostridiales;Lachnospiraceae;NA;NA; |
| a3ce47f5c5bfb0077149ad467f73284dab191abd | distal | 0.31 | 4.97E-10 | 4.57E-09 | 2.03E-09 | Bacteria;Bacteroidetes;Bacteroidia;Bacteroidales;Rikenellaceae;Alistipes;finegoldii/onderdonkii/putredinis; |
| 0a9cef5de9629f3b24f457d1ee978e28eddcc1f7 | distal | 0.29 | 0.13604218 | 0.24536179 | 0.10904805 | Bacteria;Firmicutes;Clostridia;Clostridiales;Lachnospiraceae;GCA-900066575;genus_check/stercoris; |
| 20cba8d914e27f1386fef4cc80814fbd88cfa883 | distal | 0.26 | 0.01034014 | 0.0282258 | 0.01254461 | Bacteria;Firmicutes;Clostridia;Clostridiales;Lachnospiraceae;Blautia;massiliensis; |
| 3172c57e6f43b6dd00b72d6ba94b62cd4d386f07 | distal | 0.24 | 0.24918103 | 0.39277584 | 0.17456443 | Bacteria;Firmicutes;Clostridia;Clostridiales;Lachnospiraceae;NA;NA; |
| 80cb3ba7025d47440aac3192c0673d77e72bcbb7 | distal | 0.23 | 0.65889146 | 0.76001379 | 0.33777885 | Bacteria;Proteobacteria;Gammaproteobacteria;Betaproteobacteriales;Burkholderiaceae;Sutterella;wadsworthensis; |
| d6e7fa11f119f2b038716cba3ca721b8b39871bf | distal | 0.23 | 0.139613 | 0.24738444 | 0.109947 | Bacteria;Firmicutes;Clostridia;Clostridiales;Lachnospiraceae;Coprococcus_1;catus; |
| 0694fb7ab2fc435cd49b675a4a501d5d9daa2930 | distal | 0.22 | 0.00966627 | 0.02786865 | 0.01238588 | Bacteria;Firmicutes;Clostridia;Clostridiales;Ruminococcaceae;Negativibacillus;massiliensis; |
| 3897e30a1778b4cbb87e053557cc96329d33fc01 | distal | 0.21 | 0.25372323 | 0.39277584 | 0.17456443 | Bacteria;Firmicutes;Clostridia;Clostridiales;Ruminococcaceae;Ruminiclostridium_9;NA; |
| b56b7e2dd2e753138a0a1d1e71f3056d12fed902 | distal | 0.20 | 0.10264959 | 0.18850198 | 0.0837774 | Bacteria;Firmicutes;Clostridia;Clostridiales;Lachnospiraceae;NA;NA; |
| 9416469ebfb756921565fc178646cca9ca55d008 | distal | 0.19 | 0.00152658 | 0.00550659 | 0.00244734 | Bacteria;Firmicutes;Negativicutes;Selenomonadales;Veillonellaceae;Dialister;invisus; |
| 64de730cea6eb29df43c1257bcebb75eece2daea | distal | 0.17 | 2.37E-06 | 1.41E-05 | 6.25E-06 | Bacteria;Bacteroidetes;Bacteroidia;Bacteroidales;Bacteroidaceae;Bacteroides;fragilis/xylanisolvens; |
| 0c4193ee8866c8da9694bf684014198a3870d851 | distal | 0.16 | 0.0798821 | 0.15222815 | 0.06765594 | Bacteria;Firmicutes;Clostridia;Clostridiales;Lachnospiraceae;Lachnospiraceae_NK4A136_group;NA; |
| ac7007d42a66c52ae3c2ba868adb2b2551034d4a | distal | 0.16 | 0.37414342 | 0.52484008 | 0.23325877 | Bacteria;Firmicutes;Clostridia;Clostridiales;Ruminococcaceae;Pygmaiobacter;NA; |
| 66a95ddc0a727d2990ea78674a26c3fa5d2d7f0e | distal | 0.15 | 0.00993338 | 0.02786865 | 0.01238588 | Bacteria;Firmicutes;Clostridia;Clostridiales;Lachnospiraceae;Fusicatenibacter;saccharivorans; |
| adc97521fb9230dd936e64c99f35f36ea965ff05 | distal | 0.14 | 0.00882423 | 0.02621314 | 0.01165011 | Bacteria;Bacteroidetes;Bacteroidia;Bacteroidales;Bacteroidaceae;Bacteroides;vulgatus; |
| 7b00e0e6df7ff973d165928952320aaa95e1a9a6 | distal | 0.12 | 0.3398602 | 0.49036971 | 0.21793884 | Bacteria;Firmicutes;Clostridia;Clostridiales;Ruminococcaceae;NA;NA; |
| 19a6f7454cc2c6d4c05a9f7c0790efc7641a6e08 | distal | 0.12 | 1.58E-05 | 7.58E-05 | 3.37E-05 | Bacteria;Bacteroidetes;Bacteroidia;Bacteroidales;Tannerellaceae;Parabacteroides;merdae; |
| a12bb28d688a2818753403165c417928c708839c | distal | 0.11 | 0.05326232 | 0.11207279 | 0.04980938 | Bacteria;Bacteroidetes;Bacteroidia;Bacteroidales;Bacteroidaceae;Bacteroides;cellulosilyticus/intestinalis; |
| 42adac273cd0ffae54091f9ee9abaeafafd5e365 | distal | 0.10 | 0.53437664 | 0.6746505 | 0.29984019 | Bacteria;Firmicutes;Clostridia;Clostridiales;Ruminococcaceae;Butyricicoccus;desmolans/faecihominis; |
| 7c0eb1629cbe2583bf46f6cd326d3dca259e73ce | distal | 0.10 | 0.40410954 | 0.55155491 | 0.24513185 | Bacteria;Firmicutes;Clostridia;Clostridiales;Lachnospiraceae;Blautia;luti/obeum/wexlerae; |
| c7c054e78775fd54be9ea450fca5a2cc1945d3f8 | distal | 0.06 | 0.56531582 | 0.68791444 | 0.30573518 | Bacteria;Firmicutes;Clostridia;Clostridiales;Lachnospiraceae;Tyzzerella_4;genus_check/comes; |
| db9c6b507c12df04ca9b8e7145b18122ecb99bd7 | distal | 0.06 | 0.19728854 | 0.33773123 | 0.15010053 | Bacteria;Bacteroidetes;Bacteroidia;Bacteroidales;Bacteroidaceae;Bacteroides;massiliensis/sartorii/vulgatus; |
| e6616cc3d76c4b079d8cb7ae445f9d3fc030e07f | distal | 0.06 | 0.00718523 | 0.02329593 | 0.01035359 | Bacteria;Bacteroidetes;Bacteroidia;Bacteroidales;Bacteroidaceae;Bacteroides;vulgatus; |
| b76e229e5444427d2ec5554d0665d25c8dace5ca | distal | 0.05 | 0.80230808 | 0.87132383 | 0.38724924 | Bacteria;Firmicutes;Clostridia;Clostridiales;Lachnospiraceae;Lachnoclostridium;urinimassiliense; |
| 2b0c96c19e80e9f5a838843afb0b6c138d7172f7 | distal | 0.05 | 0.6249831 | 0.7465173 | 0.33178051 | Bacteria;Firmicutes;Clostridia;Clostridiales;Ruminococcaceae;Ruminiclostridium_9;NA; |
| 90882810d9fea2292cdeaafc923ba449596878f4 | distal | 0.04 | 0.66219023 | 0.76001379 | 0.33777885 | Bacteria;Proteobacteria;Gammaproteobacteria;Betaproteobacteriales;Burkholderiaceae;Parasutterella;excrementihominis; |
| 66f19525c1c8f7362b552914ce5fbe9e48d50b46 | distal | 0.04 | 0.03656916 | 0.08394285 | 0.03730738 | Bacteria;Bacteroidetes;Bacteroidia;Bacteroidales;Bacteroidaceae;Bacteroides;uniformis; |
| 56898b08756f5cc0037d3f46cc1d66049179c146 | distal | 0.03 | 0.54838047 | 0.67544424 | 0.30019295 | Bacteria;Firmicutes;Negativicutes;Selenomonadales;Acidaminococcaceae;Phascolarctobacterium;faecium; |
| 3117ba47798f82c79f4f389bba3d2ba4a248922f | distal | 0.02 | 0.77679126 | 0.85278171 | 0.37900843 | Bacteria;Bacteroidetes;Bacteroidia;Bacteroidales;Bacteroidaceae;Bacteroides;fragilis/ovatus; |
| a7266616bf8e0fbf8eb72fe0988c33ef323183a1 | distal | 0.02 | 0.88728945 | 0.92387871 | 0.41060662 | Bacteria;Proteobacteria;Gammaproteobacteria;Betaproteobacteriales;Burkholderiaceae;Parasutterella;NA; |
| 53369c6d2bfa8ad07dfda61d76f3d3dd118b6749 | distal | 0.02 | 0.8355821 | 0.8883557 | 0.39481885 | Bacteria;Firmicutes;Clostridia;Clostridiales;Lachnospiraceae;Lachnoclostridium;NA; |
| 3e6bd7ced29d1f765540677d259298edea179491 | distal | 0.00 | 0.9846399 | 0.99993574 | 0.44440924 | Bacteria;Firmicutes;Clostridia;Clostridiales;Ruminococcaceae;Flavonifractor;plautii; |
| c8e94768a9e3f9d30397b41f123b5e5ace35d022 | proximal | -0.01 | 0.92982684 | 0.95829092 | 0.42590071 | Bacteria;Bacteroidetes;Bacteroidia;Bacteroidales;Barnesiellaceae;Barnesiella;intestinihominis; |
| 043c07d541da5b44bbf8c45a7973a4790146fef6 | proximal | -0.01 | 0.84665641 | 0.89075309 | 0.39588434 | Bacteria;Bacteroidetes;Bacteroidia;Bacteroidales;Bacteroidaceae;Bacteroides;massiliensis; |
| 55013390e079b6ea791b2deeecf94c1787accb20 | proximal | -0.02 | 0.81844639 | 0.87939453 | 0.39083617 | Bacteria;Proteobacteria;Gammaproteobacteria;Betaproteobacteriales;Burkholderiaceae;Parasutterella;excrementihominis; |
| eb6dc9f905c574e9ba6937eb007f0663e85ebf0f | proximal | -0.03 | 0.69869905 | 0.78409559 | 0.34848172 | Bacteria;Bacteroidetes;Bacteroidia;Bacteroidales;Rikenellaceae;Alistipes;finegoldii/onderdonkii; |
| a7b03011c8c401c5e93b8aafb29af25488640436 | proximal | -0.05 | 0.35444821 | 0.50421506 | 0.22409223 | Bacteria;Bacteroidetes;Bacteroidia;Bacteroidales;Bacteroidaceae;Bacteroides;caccae; |
| 9da7cb253ff6f8eb72c6f8c8212e187a60380547 | proximal | -0.05 | 0.52432382 | 0.67033804 | 0.29792357 | Bacteria;Firmicutes;Clostridia;Clostridiales;Lachnospiraceae;Lachnospira;genus_check/eligens; |
| 92a9e6fd06b63cc48625058e4125b1a2f2cf3048 | proximal | -0.07 | 0.42963815 | 0.56355134 | 0.25046352 | Bacteria;Bacteroidetes;Bacteroidia;Bacteroidales;Bacteroidaceae;Bacteroides;nordii; |
| 1a5db7cbf3e568848481d107f6f2da7e423425c1 | proximal | -0.07 | 0.41920414 | 0.55710024 | 0.2475964 | Bacteria;Bacteroidetes;Bacteroidia;Bacteroidales;Marinifilaceae;Odoribacter;splanchnicus; |
| 0c2b3a10e2af94b962ba7528f751204d55900582 | proximal | -0.07 | 0.01718097 | 0.04566521 | 0.02029535 | Bacteria;Firmicutes;Clostridia;Clostridiales;Ruminococcaceae;Faecalibacterium;prausnitzii; |
| 96403712f55e764efc66be5f1a596c371dba80da | proximal | -0.08 | 0.16211253 | 0.28229941 | 0.12546453 | Bacteria;Firmicutes;Clostridia;Clostridiales;Lachnospiraceae;Lachnospiraceae_UCG-010;NA; |
| 6c63c7c4b65e18ecb6da353513b0dc9620820947 | proximal | -0.09 | 0.52405136 | 0.67033804 | 0.29792357 | Bacteria;Firmicutes;Clostridia;Clostridiales;Ruminococcaceae;Oscillibacter;NA; |
| 5d2cfcaf12bdc5e5bef0b38e87406831f02c21ab | proximal | -0.10 | 0.38202849 | 0.52855996 | 0.23491203 | Bacteria;Bacteroidetes;Bacteroidia;Bacteroidales;Tannerellaceae;Parabacteroides;distasonis; |
| 8bac4e22d77ae1efc469563bab275f452e002119 | proximal | -0.10 | 0.09997194 | 0.18698455 | 0.083103 | Bacteria;Bacteroidetes;Bacteroidia;Bacteroidales;Marinifilaceae;Odoribacter;splanchnicus; |
| cbe56d8d19aa6175f4664cbf2bc0532c68243477 | proximal | -0.11 | 0.02342382 | 0.05770259 | 0.02564521 | Bacteria;Firmicutes;Clostridia;Clostridiales;Ruminococcaceae;Faecalibacterium;prausnitzii; |
| d0fcb4043b758d8c8b416265dce6280d2bc137a7 | proximal | -0.15 | 0.2566654 | 0.39277584 | 0.17456443 | Bacteria;Firmicutes;Clostridia;Clostridiales;Lachnospiraceae;Lachnospiraceae_ND3007_group;NA; |
| dbd5144f31c5e2f56f5f6b4459bfc7075479e16e | proximal | -0.15 | 1.99E-05 | 9.11E-05 | 4.05E-05 | Bacteria;Bacteroidetes;Bacteroidia;Bacteroidales;Bacteroidaceae;Bacteroides;vulgatus; |
| b75db79d71e49907faca3540adab49d1720f69f7 | proximal | -0.15 | 1.82E-30 | 6.12E-29 | 2.72E-29 | Bacteria;Bacteroidetes;Bacteroidia;Bacteroidales;Bacteroidaceae;Bacteroides;dorei/vulgatus; |
| 589c1547cb93f3daa004788a3e8186b40b3a312e | proximal | -0.20 | 0.25517328 | 0.39277584 | 0.17456443 | Bacteria;Firmicutes;Clostridia;Clostridiales;Lachnospiraceae;Blautia;faecis; |
| c6a658b20410df6df7f5cefcab3fb9decece9b76 | proximal | -0.22 | 0.2398753 | 0.39076461 | 0.17367056 | Bacteria;Firmicutes;Clostridia;Clostridiales;Ruminococcaceae;DTU089;NA; |
| a882b133d4083f6fcc2ae50bd14dbe3d5446bb24 | proximal | -0.23 | 0.00015252 | 0.00064187 | 0.00028527 | Bacteria;Bacteroidetes;Bacteroidia;Bacteroidales;Bacteroidaceae;Bacteroides;dorei/sartorii/vulgatus; |
| 249bed28190c2a1fa1b808457c4bb15912e4722d | proximal | -0.23 | 0.03870701 | 0.08687573 | 0.03861086 | Bacteria;Firmicutes;Clostridia;Clostridiales;Lachnospiraceae;NA;NA; |
| fd1b56f2f52050a8575af68b53b5a691bea5db8c | proximal | -0.27 | 0.02735738 | 0.065788 | 0.02923867 | Bacteria;Firmicutes;Clostridia;Clostridiales;Ruminococcaceae;Negativibacillus;NA; |
| 26bf8c066146b41167a37935f3f358101696b6ee | proximal | -0.27 | 0.02182281 | 0.0551026 | 0.02448968 | Bacteria;Firmicutes;Erysipelotrichia;Erysipelotrichales;Erysipelotrichaceae;Holdemania;filiformis; |
| e4bb49ce45f25c04d029c0f3c2a02ae1f780ed7e | proximal | -0.28 | 5.88E-19 | 8.49E-18 | 3.77E-18 | Bacteria;Bacteroidetes;Bacteroidia;Bacteroidales;Bacteroidaceae;Bacteroides;massiliensis; |
| 630f4d47a9b05aa8f65f112233ab096262308e5b | proximal | -0.31 | 3.74E-21 | 7.55E-20 | 3.35E-20 | Bacteria;Bacteroidetes;Bacteroidia;Bacteroidales;Bacteroidaceae;Bacteroides;massiliensis; |
| 9f1cd9232177f8118fc68906e04b28bcd454311b | proximal | -0.34 | 0.04879004 | 0.10484669 | 0.04659783 | Bacteria;Firmicutes;Clostridia;Clostridiales;Ruminococcaceae;Ruminococcaceae_UCG-013;NA; |
| d7df7ac38235e7bfd8f7509a774a0023ff193d2a | proximal | -0.36 | 0.0592864 | 0.11975852 | 0.05322522 | Bacteria;Firmicutes;Clostridia;Clostridiales;Lachnospiraceae;Lachnoclostridium;NA; |
| 6bfa0df47a7273a5e30dbefaa981330bd1d5dad4 | proximal | -0.39 | 0.01866509 | 0.0483378 | 0.02148314 | Bacteria;Firmicutes;Clostridia;Clostridiales;Ruminococcaceae;Butyricicoccus;desmolans/faecihominis; |
| 2b2000704aa739ea00a45a8bed5bdd42c8cafcb8 | proximal | -0.41 | 0.40978072 | 0.55183803 | 0.24525768 | Bacteria;Firmicutes;Clostridia;Clostridiales;Lachnospiraceae;NA;NA; |
| 1910ca1b089d8231b2e67f8454cdbd5c020c3144 | proximal | -0.41 | 7.22E-11 | 7.29E-10 | 3.24E-10 | Bacteria;Firmicutes;Erysipelotrichia;Erysipelotrichales;Erysipelotrichaceae;Erysipelatoclostridium;ramosum; |
| 407411bafacade3dea8e72c2ec6880198037a6df | proximal | -0.42 | 0.29555209 | 0.44553375 | 0.19801204 | Bacteria;Firmicutes;Clostridia;Clostridiales;Ruminococcaceae;Anaerotruncus;colihominis; |
| 66cc8012b26068af882ad952e91345d0956686bc | proximal | -0.43 | 0.0650271 | 0.12877916 | 0.05723433 | Bacteria;Firmicutes;Clostridia;Clostridiales;Ruminococcaceae;Ruminiclostridium_5;NA; |
| b0e67ad37c689fd848f4ac9b923029d9c5b97b84 | proximal | -0.45 | 2.44E-18 | 3.08E-17 | 1.37E-17 | Bacteria;Firmicutes;Clostridia;Clostridiales;Lachnospiraceae;Roseburia;inulinivorans; |
| aba2338cfa6a47bbec11e832e4034f127329942f | proximal | -0.46 | 0.00096425 | 0.00360701 | 0.00160309 | Bacteria;Firmicutes;Clostridia;Clostridiales;Lachnospiraceae;CAG-56;NA; |
| f2701317ac41eafe7aaee21a85664470b6b56cde | proximal | -0.49 | 0.00029402 | 0.00114216 | 0.00050762 | Bacteria;Bacteroidetes;Bacteroidia;Bacteroidales;Bacteroidaceae;Bacteroides;eggerthii; |
| ab05bcb443299370fc72115db48e6b4157a86442 | proximal | -0.64 | 0.05569779 | 0.11480565 | 0.05102397 | Bacteria;Firmicutes;Clostridia;Clostridiales;Ruminococcaceae;NA;NA; |
| afb8924308524d3194cd3aefe538773646df563d | proximal | -0.76 | 0.00023374 | 0.00094431 | 0.00041969 | Bacteria;Firmicutes;Clostridia;Clostridiales;Ruminococcaceae;Ruminococcaceae_UCG-004;NA; |
| 647fb0235ac545a07e5ed6c880b2318e6f45df29 | proximal | -0.76 | 8.10E-09 | 5.84E-08 | 2.60E-08 | Bacteria;Bacteroidetes;Bacteroidia;Bacteroidales;Bacteroidaceae;Bacteroides;faecis/thetaiotaomicron; |
| e9c75d4517ae2c00d0cc63923bece93188d46d6d | proximal | -0.81 | 0.72698484 | 0.80687328 | 0.35860499 | Bacteria;Bacteroidetes;Bacteroidia;Bacteroidales;Bacteroidaceae;Bacteroides;vulgatus; |
| c424e8f38ff520f51b49c7c71bc434b99420f16b | proximal | -0.90 | 0.54602421 | 0.67544424 | 0.30019295 | Bacteria;Firmicutes;Clostridia;Clostridiales;Lachnospiraceae;Lachnospira;pectinoschiza; |
| e7f32edb580c105327d53c5e28250b00537e62ce | proximal | -1.54 | 9.05E-10 | 7.62E-09 | 3.39E-09 | Bacteria;Fusobacteria;Fusobacteriia;Fusobacteriales;Fusobacteriaceae;Fusobacterium;nucleatum; |
| 9aabaca70ab3616bcc40b4e2e09a6d024049e7ee | proximal | -1.85 | 0.04012358 | 0.08809742 | 0.03915382 | Bacteria;Firmicutes;Bacilli;Lactobacillales;Streptococcaceae;Streptococcus;salivarius/thermophilus; |
| df4cfc980121095e2626e4d4f42c03fdd4c8428e | proximal | -4.27 | 0.00853199 | 0.02611306 | 0.01160563 | Bacteria;Fusobacteria;Fusobacteriia;Fusobacteriales;Fusobacteriaceae;Fusobacterium;nucleatum; |
| fa168b426edd535933f3a3828a6b828df49062a5 | proximal | -29.53 | 0.99993574 | 0.99993574 | 0.44440924 | Bacteria;Epsilonbacteraeota;Campylobacteria;Campylobacterales;Campylobacteraceae;Campylobacter;gracilis; |

**Table S7:** Differentially abundant microbes in individuals with dMMR CRC from an independent cohort. Blue boxes highlight microbes enriched in tumor tissue samples as compared to normal adjacent samples.

| OTU_Id | baseMean | log2FoldChange | lfcSE | stat | pvalue | padj | Phylum | Class | Order | Family | Genus | Species |
|---|---|---|---|---|---|---|---|---|---|---|---|---|
| 1139840 | 147.795 | 3.273 | 0.835 | 3.920 | 0.000 | 0.008 | Spirochaetes | Spirochaetes | Spirochaetales | Spirochaetaceae | Treponema | |
| 1065387 | 36.266 | 2.079 | 0.972 | 2.138 | 0.032 | 0.372 | Bacteroidetes | Bacteroidia | Bacteroidales | [Paraprevotellaceae] | [Prevotella] | |
| 1096339 | 239.138 | 2.007 | 0.888 | 2.261 | 0.024 | 0.372 | Fusobacteria | Fusobacteriia | Fusobacteriales | Fusobacteriaceae | Fusobacterium | |
| 128205 | 222.417 | 1.800 | 0.837 | 2.150 | 0.032 | 0.372 | Bacteroidetes | Bacteroidia | Bacteroidales | Bacteroidaceae | Bacteroides | fragilis |
| 1004910 | 68.434 | 1.501 | 0.712 | 2.109 | 0.035 | 0.372 | Bacteroidetes | Bacteroidia | Bacteroidales | Prevotellaceae | Prevotella | |
| 144395 | 18.615 | -1.662 | 0.762 | -2.181 | 0.029 | 0.372 | Firmicutes | Clostridia | Clostridiales | Lachnospiraceae | Dorea | |
| 1551841 | 49.989 | -2.234 | 0.936 | -2.387 | 0.017 | 0.372 | Firmicutes | Clostridia | Clostridiales | Lachnospiraceae | [Ruminococcus] | gnavus |
| 1012372 | 32.916 | -2.724 | 1.016 | -2.682 | 0.007 | 0.311 | Firmicutes | Erysipelotrichi | Erysipelotrichales | Erysipelotrichaceae | | |

**Table S8**: Differentially abundant microbes in individuals with pMMR CRC from an independent cohort. Blue boxes highlight microbes enriched in tumor tissue samples as compared to normal adjacent samples.

| OTU_Id | baseMean | log2FoldChange | lfcSE | stat | pvalue | padj | Phylum | Class | Order | Family | Genus | Species |
|---|---|---|---|---|---|---|---|---|---|---|---|---|
| 106952 | 37.452 | 2.081 | 0.604 | 3.443 | 0.001 | 0.014 | Proteobacteria | Gammaproteobacteria | Oceanospirillales | Halomonadaceae | Candidatus Portiera | aleyrodidarum |
| 135755 | 22.419 | 1.879 | 0.551 | 3.412 | 0.001 | 0.014 | Proteobacteria | Gammaproteobacteria | HTCC2188 | HTCC2089 | | |
| 1047065 | 10.921 | 1.854 | 0.496 | 3.735 | 0.000 | 0.008 | Proteobacteria | Alphaproteobacteria | Rhodobacterales | Rhodobacteraceae | | |
| 4357652 | 17.705 | 1.798 | 0.653 | 2.755 | 0.006 | 0.031 | Firmicutes | Clostridia | Clostridiales | Clostridiaceae | Alkaliphilus | |
| 1106195 | 16.367 | 1.769 | 0.546 | 3.236 | 0.001 | 0.018 | OD1 | ZB2 | | | | |
| 609704 | 19.632 | 1.634 | 0.574 | 2.847 | 0.004 | 0.031 | Proteobacteria | Gammaproteobacteria | Enterobacteriales | Enterobacteriaceae | Providencia | |
| 1134730 | 22.018 | 1.535 | 0.548 | 2.799 | 0.005 | 0.031 | Proteobacteria | Gammaproteobacteria | Oceanospirillales | Halomonadaceae | Candidatus Portiera | |
| 103288 | 9.933 | 1.503 | 0.491 | 3.063 | 0.002 | 0.025 | Actinobacteria | Actinobacteria | Actinomycetales | | | |
| 778029 | 23.263 | 1.485 | 0.651 | 2.281 | 0.023 | 0.087 | Chlorobi | BSV26 | C20 | | | |
| 1546868 | 11.248 | 1.464 | 0.529 | 2.768 | 0.006 | 0.031 | Acidobacteria | [Chloracidobacteria] | RB41 | | | |
| 101584 | 147.234 | 1.432 | 0.366 | 3.909 | 0.000 | 0.008 | Bacteroidetes | Bacteroidia | Bacteroidales | | | |
| 1135525 | 10.774 | 1.422 | 0.442 | 3.220 | 0.001 | 0.018 | OD1 | | | | | |
| 1010721 | 19.395 | 1.417 | 0.618 | 2.291 | 0.022 | 0.087 | Bacteroidetes | Flavobacteriia | Flavobacteriales | [Weeksellaceae] | Chryseobacterium | |
| 1108726 | 54.398 | 1.398 | 0.604 | 2.316 | 0.021 | 0.087 | Proteobacteria | Betaproteobacteria | Burkholderiales | Comamonadaceae | Methylibium | |
| 1115384 | 257.797 | 1.322 | 0.434 | 3.045 | 0.002 | 0.025 | Planctomycetes | Planctomycetia | Pirellulales | Pirellulaceae | | |
| 1028553 | 14.510 | 1.190 | 0.483 | 2.465 | 0.014 | 0.069 | Planctomycetes | Planctomycetia | Planctomycetales | Planctomycetaceae | Planctomyces | |
| 1016598 | 208.568 | -0.705 | 0.311 | -2.265 | 0.024 | 0.087 | Firmicutes | Clostridia | Clostridiales | Ruminococcaceae | | |
| 110562 | 54.305 | -1.089 | 0.366 | -2.973 | 0.003 | 0.028 | Firmicutes | Clostridia | Clostridiales | Ruminococcaceae | Ruminococcus | |
| 147702 | 56.471 | -1.133 | 0.465 | -2.434 | 0.015 | 0.071 | Firmicutes | Clostridia | Clostridiales | Ruminococcaceae | Faecalibacterium | prausnitzii |
| 1138850 | 22.615 | -1.276 | 0.450 | -2.835 | 0.005 | 0.031 | Firmicutes | Clostridia | Clostridiales | Lachnospiraceae | Coprococcus | |
| 195508 | 6.569 | -1.352 | 0.596 | -2.270 | 0.023 | 0.087 | Bacteroidetes | Bacteroidia | Bacteroidales | Bacteroidaceae | Bacteroides | caccae |
| 1067143 | 62.681 | -1.563 | 0.562 | -2.784 | 0.005 | 0.031 | Bacteroidetes | Bacteroidia | Bacteroidales | Rikenellaceae | | |
| 1733364 | 22.419 | -1.596 | 0.549 | -2.908 | 0.004 | 0.031 | Bacteroidetes | Bacteroidia | Bacteroidales | Bacteroidaceae | Bacteroides | uniformis |
